# What Does the General Public Know (or Not) About Neuroscience? Effects of Age, Region and Profession in Brazil

**DOI:** 10.3389/fnhum.2022.798967

**Published:** 2022-03-04

**Authors:** Analía Arévalo, Estefania Simoes, Fernanda Petinati, Guilherme Lepski

**Affiliations:** ^1^Division of Functional Neurosurgery, Department of Psychiatry, Medical School, University of São Paulo, São Paulo, Brazil; ^2^Cancer Metabolism Research Group, Cell and Developmental Biology, University of São Paulo, São Paulo, Brazil; ^3^Psychotherapy Department, Institute of Psychiatry, University of São Paulo, São Paulo, Brazil; ^4^Department of Neurosurgery, Eberhard Karls University of Tübingen, Tübingen, Germany

**Keywords:** neuromyths, neuroscience, public knowledge, education, higher learning

## Abstract

The field of Neuroscience has experienced a growing interest in recent decades, which has led to an exponential growth in the amount of related information made available online as well as the market for Neuroscience-related courses. While this type of knowledge can be greatly beneficial to people working in science, health and education, it can also benefit individuals in other areas. For example, neuroscience knowledge can help people from all fields better understand and critique information about new discoveries or products, and even make better education- and health-related decisions. Online platforms are fertile ground for the creation and spread of fake information, including misrepresentations of scientific knowledge or new discoveries (e.g., neuromyths). These types of false information, once spread, can be difficult to tear down and may have widespread negative effects. For example, even scientists are less likely to access retractions of peer-reviewed articles than the original discredited articles. In this study we surveyed general knowledge about neuroscience and the brain among volunteers in Brazil, Latin America’s largest country. We were interested in evaluating the prevalence of neuromyths in this region, and test whether knowledge/neuromyth endorsement differs by age, region, and/or profession. To that end, we created a 30-item survey that was anonymously answered online by 1128 individuals. While younger people (20–29-year-olds) generally responded more accurately than people 60 and older, people in the North responded significantly worse than those in the South and Southeast. Most interestingly, people in the biological sciences consistently responded best, but people in the health sciences responded no better than people in the exact sciences or humanities. Furthermore, years of schooling did not correlate with performance, suggesting that quantity may surpass quality when it comes to extension or graduate-level course offerings. We discuss how our findings can help guide efforts toward improving access to quality information and training in the region.

## Introduction

The field of neuroscience has significantly grown worldwide in the last few decades. Interestingly, since the 1990s (known in the United States as the Decade of the Brain), interest in and the pursuit of knowledge in this field have only seemed to grow (OECD, 2002; Dekker et al., 2012). According to PubMed, in the mid 1960’s, an average of 3,000 articles including the word “brain” were published per year; in 2019, this number increased to 94,615 (Markram, 2013; Fan and Markram, 2019; Tokuhama-Espinosa, 2019).

Neuroscience-related topics represent critical general knowledge and information in modern society and are therefore relevant for a wide range of professions and lifestyles. Among other things, neuroscientific knowledge can help one learn faster, read better, acquire motor or sports-related abilities, improve quality of sleep, increase concentration, and stabilize one’s emotions (Landi et al., 2013; Stanley and Krakauer, 2013; Dubinsky et al., 2019; Humeau et al., 2019; Klinzing et al., 2019; van Kesteren and Meeter, 2020). It can also help educators improve their teaching strategies and learners improve their performance, which can in turn orient important educational and health policies (OECD, 2002; Goswami, 2006; Howard-Jones, 2014; Dubinsky et al., 2019). Critically, neuroscience-related knowledge can help prevent discrimination in society, by eliminating old inaccurate views regarding biological differences among genders, races, or cultural or socioeconomic groups.

Among the first 50 result pages of a simple Google search conducted in July of 2020, we found more than 400 free/open courses in Neuroscience or Neuroeducation offered in Brazil. This high number of free courses suggests a growing interest among Brazilians in pursuing academic training in Neuroscience-related areas. Almost all the courses we found are offered online for free or at an affordable cost. Furthermore, the average number of hours required for course completion is a mere 24 (maximum 80), which may arguably not be enough time to gain expertise (but see Darling-Hammond et al., 2011). Most interestingly, less than 10% of these courses were associated with an accredited higher learning institution, making it difficult to determine the quality of the content offered. Among longer graduate-level extension courses, only 11 (less than 3%) are offered by universities that are well placed in the Ministry of Education’s (MEC) most recent general course index (IGC, 2018). Reduced cost and time investment may be attractive features when choosing a course, particularly when consumers have little access to reliable reviews or evaluations of the countless products available. In sum, there is a great supply of courses for an increasing demand, but it is difficult to assess the quality or effectiveness of these courses.

While scientists in any part of the world are trained to analyze and critique information (scientific or otherwise), through people rely mostly on big media or online venues for access to new research, theories and discoveries. But what kinds of scientific information can laypeople access though these sources? In Brazil, several companies and portals translate scientific research to lay language, but this is often done by non-specialized journalists. Currently, there are more than 31,306 communication companies in Brazil (Grupo de Mídia São Paulo, 2019), and approximately 16,477 online portals (We are Social, 2020).

While in other countries such as the United States, people have also reported using the internet as their main source of information (Zambo and Zambo, 2009), these numbers are even higher in Brazil. Globally, 59% of people have access to the internet, and 49% of those people use at least one social media platform. In Brazil, these numbers are 66% and 71%, respectively. Also, while average internet use per day worldwide is 6 h and 42 min, average daily use in Brazil is 9 h and 29 min, with 85% of Brazilians going online on a daily basis (We are Social, 2020).

The recent phenomenon of Fake News has largely contributed to the public’s general misinformation regarding healthcare knowledge (Merchant and Asch, 2018), which could have long-lasting negative effects, especially when such misinformation infiltrates areas such as basic education. Misinformation can lead to misguided educational methods that could affect generations to come (Peters, 2018) and negatively impact decision-making, driving entire communities toward choices that are not scientifically sound (Scheufele and Krause, 2019), such as anti-vaccination movements (Chiou and Tucker, 2018) or the endorsement of inappropriate medical treatments (Lavorgna et al., 2018). In 2018, Brazil’s Ministry of Health launched a secure WhatsApp line to answer people’s questions regarding online information; the report they provided at the end of one year revealed that 77% of questions answered were based on fake news^1^.

Brazil is a country of continental proportions, in terms of size and in the number of different cultures, socioeconomic levels and methods of information consumption (IBGE, 2021). In order to take on the challenge of improving the quality of available information and training (both inside and outside academic settings), one must understand where the biggest problems lie. To this end, we used the most searched neuroscience-related terms and questions to create a survey of 30 true/false statements which we distributed among our personal and professional contacts all over Brazil and published on several social media platforms. We obtained anonymous responses from 1128 individuals representing different age groups, regions within Brazil and professions.

Our motivation for assessing whether profession made a difference in performance was to see whether assumptions about knowledge among people in different fields were upheld (e.g., people in health or science should know more about neuroscience than people in the humanities or the exact sciences). Similarly, we wanted to investigate whether people in different regions performed differently because regions in Brazil are unequally favored in terms of wealth, resources and education: total years of schooling are significantly higher and illiteracy rates are significantly lower in the South and Southeast relative to the North and Northeast (IBGE, 2021). A study conducted by the Brazilian Institute for Geography and Statistics (Instituto Brasileiro de Geografia e Estatística, IBGE) in 2018 revealed that internet use is highest among 18–29-year-olds (90–91%), and lowest among individuals 60 and older (38.7%), with steadily declining numbers as age increases (IBGE, 2018). Thus, given that much information (accurate or inaccurate) is obtained from the internet (Markram, 2013), and internet use is not equal across age groups, we questioned whether performance would also vary among the age groups tested.

**FIGURE 1:**
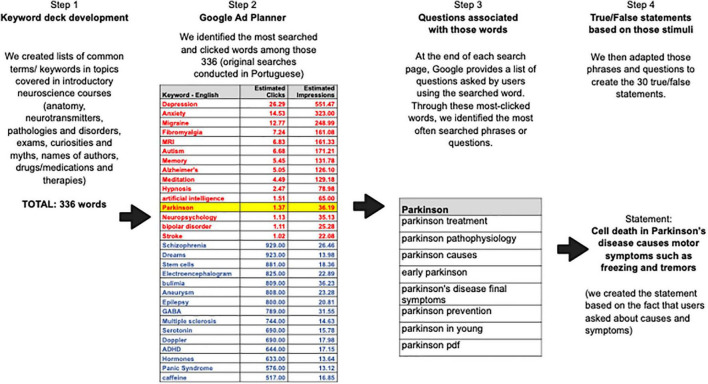
Flowchart showing the steps taken to create the survey.

In terms of specific answers, we expected better performance on statements regarding vaccines and disorders (e.g., Alzheimer’s Disease, Parkinson’s Disease, autism and epilepsy), as these are often covered in the media. We also expected relatively good performance on statements about child development, which is also widely publicized and discussed (on YouTube channels, blogs and social media platforms). In contrast, we expected lower performance on common neuromyths (e.g., brain size and intelligence, differences between female and male brains), see Figure 1 for a graphic illustration of this 4-step process as these are often perpetuated in films and TV (among other sources), and on more complex topics requiring a deeper understanding of neuroscience (e.g., neuronal function, systems neuroscience), as these concepts are more complex and are usually covered in formal courses.

Our goal was to obtain a clearer picture of general knowledge across these groups, including specific knowledge gaps, as well as which neuromyths are endorsed the most. In the short term, this information can help guide new and better ways of improving scientific communication. In the long term, this information could motivate the development of better-quality courses (e.g., undergraduate, graduate, extension, and free). For survey participants who expressed interest, we made available a document containing the answers and explanations for each statement written in simple lay terms, as a small initial contribution to the spread of science-based information (see Supplementary Figure 1).

## Materials and Methods

### Procedure

In a pilot study conducted by our group in 2018, 401 participants anonymously responded to a Google survey containing questions and statements about general knowledge in neuroscience, which we created based on previous publications in the field and on conversations with colleagues and students. All four authors of this article are closely involved with a multi-professional extension course in neuroscience offered at University of São Paulo’s Medical School (two are the course’s coordinators and two are ex-students); some of the questions were created based on students’ questions asked at the beginning of that course. Those pilot data were not published because we noticed a number of flaws with the way some questions were presented (e.g., unclear wording), but it did help us identify valid questions and contributed to the creation of the subsequent survey (see below). For example, in one open answer question, participants were asked what they would ask a neuroscientist if they were to meet one at a party. The answers to that question provided us with important insight into individuals’ doubts and curiosities.

Then, in 2019, we took on the challenge one more time. However, this time, we used a different approach.

Step 1: Each of the authors created lists of common terms or keywords in the following areas commonly covered in introductory neuroscience courses: anatomy, neurotransmitters, pathologies and disorders, exams, curiosities and myths, drugs/medications and therapies. This yielded a total of 336 words (see Supplementary Table 1).
Step 2: We then inserted those words into the Keyword Planner within Google Ads (Google’s tool for creating advertisements on Google’s platform and networks) and identified the number of searches and clicks for those words in Brazil for an entire year (between early 2018 and April 2019). This allowed us to identify the 15 keywords with the largest click volume in Brazil within that period.
Step 3: Next, we conducted simple Google searches using those 15 words (and an additional 15 words chosen from the pilot study described above) to identify the questions most often associated with those keywords within searches. In other words, through these most-clicked words, we were able to identify the most often searched phrases or questions (see Supplementary Table 2), which we then used to create the 30 true/false statements. Basically, our stimuli were adaptations of the questions we identified (Step 4).

The survey was administered via Google Surveys and was made available on the authors’ and colleagues’ social media platforms between September 9*^th^* and October 16*^th^*, 2019. It was also distributed to colleagues, students, friends and family in all five regions of Brazil, who in turn shared with their own personal and professional networks.

Order of presentation was balanced to avoid clusters of true or false answers or similar themes and all participants viewed all 30 statements in the same order. Only after answering each question could participants view and answer the following question. Table 1 lists all 30 statements, overall response accuracy for each, and whether answers varied by age, region or profession.

**TABLE 1:** Number of participants in each region and percent of total.

Region	Number of participants	% of total
North	44	3.90
Northeast	154	13.65
Midwest	41	3.63
Southeast	756	67.02
South	109	9.66
Not declared	24	2.13
TOTAL	1128	100

The study was carried out in accordance with the Declaration of Helsinki. While ethical compliance varies across countries and institutions, online questionnaires to unidentified adults generally do not require IRB approval, which was the case at our institutions. In line with the *Ethical Standards of the American Educational Research Association* (Strike et al., 2002), the recommendations for good practice in designing internet-based research (Gupta, 2017), and *Mixed Methods Research Methodologies* (Terrell, 2012), or our online survey, we were transparent in recruiting, considered participant privacy and ensured secure communication protocols, obtained informed consent, allowed participants the opportunity to withdraw from the research at any time, and did not subsequently use the data for unethical practices. We also explained the study’s purpose, indicated that anonymity would be protected at all times by never collecting (or storing) name or any other identifying information, and coding answers so that these could not be associated with a particular participant. The first page of the survey explained these issues and asked participants whether they agreed with their anonymous answers being used in the research study. Answers from those who did not agree were excluded from the database before analyses were conducted.

### Participants

A total of 1128 individuals provided online anonymous answers to the entire survey and provided information regarding age (10–60+), profession (biological sciences, exact sciences, humanities, health sciences, retired, not working, or other) and region (South, Southeast, Midwest, North, or Northeast). These questions were presented in a multiple-choice format. Also, in a free answer format, they were asked to indicate their last (completed or incomplete) level of schooling (from grammar school to post-doctoral work), the number of years in their declared profession, and total number of years of education (as this can vary in Brazil even within the same degree). All surveys that were completed in their entirety and submitted (*n* = 1128) and for which participants gave consent, were included in the analyses.

In terms of age, 34% (*n* = 380) of participants were in the 30–39 group, 24% (*n* = 266) were in the 20–29 group, 21% (*n* = 236) were in the 40–49 group, 12% (*n* = 137) were in the 50–59 group, 6% were in the oldest group (60 or older; *n* = 68), and 4% were in the youngest group (10–19 years old; *n* = 41) (Table 2).

**TABLE 2:** Number of participants in each age group and percent of total.

AGE groups (in years)	Number of participants	% of total
10–19	41	3.63
20–29	266	23.58
30–39	380	33.69
40–49	236	20.92
50–59	137	12.15
60 and older	68	6.03
TOTAL	1128	100

In terms of Brazilian regions, most participants were from the Southeast (*n* = 756; 67%), followed by the Northeast (*n* = 154; 14%), South (*n* = 109; 10%), North (*n* = 44; 4%) and Midwest (*n* = 41; 4%). Twenty-four participants (2%) did not declare region and were thus excluded from analyses based on this variable (Table 1).

In terms of profession, 36% (*n* = 405) of respondents declared studying or working in the humanities, 27% (*n* = 307) in the health sciences, 9% (*n* = 104) in the exact sciences, 8% (*n* = 92) in biological sciences, and 20% (*n* = 220) declared other, retired, or not working (Table 3).

**TABLE 3:** Number of participants in each profession and percent of total.

Profession	Number of participants	% of total
Biological sciences	92	8.16
Exact sciences	104	9.22
Humanities	405	35.90
Health sciences	307	27.22
Other/Retired/Not working	220	19.50
TOTAL	1128	100

While we had no way of controlling the number of participants that would respond from each region as responses were entirely voluntary (and we used a snowball sampling method), the number of respondents from each region was strikingly proportional to national regional populations. Table 4 below shows population by region in Brazil and in the current study, as well as the percentage each sample represents of the larger population. A chi-squared test revealed that the regional distributions did not differ significantly between our study and the total population (all chi-square *p*s > 0.05; see Table 4 for chi-square values).

**TABLE 4:** Population by region in Brazil (IBGE, 2021) and in our study (n and % of total for each).

	Brazilian Population		Our study		Chi-square	p-value
	n	% of total	n	% of total		
Southeast	89,632.91	42%	756	67%	3.01	*p* > 0.5
Northeast	57,667.84	27%	154	14%	2.01	*p* > 0.5
South	30,402.59	14%	109	10%	0.32	*p* > 0.5
North	18,906.96	9%	44	4%	0.94	*p* > 0.5
Midwest	16,707.34	8%	41	4%	0.65	*p* > 0.5
Undeclared			23	2%		
Total Brazil	213,317.64	100%	100%	100%		

*Chi-squared and associated p values revealing the regional distribution did not differ significantly between groups.*

### Survey

The 30 statements that made up the survey were viewed and responded by all participants in the same order (see Table 5).

**TABLE 5:** Survey.

Question	CA	%	Age	Region	Prof	SC
1. Despite weighing approximately 1.2 kg and having between 80 and 100 billion neurons, we only use 10% of our brain’s capacity	F	45	*p* < 0.0001		*p* < 0.0001	NM
2. Structural differences between male and female brains are so obvious that any professional can identify a person’s gender simply by looking at an image of their brain	F	17			*p* < 0.0001	NM
3. Alzheimer’s disease can only be diagnosed after death. In life, behaviors can be identified through neuropsychological tests that suggest the presence of the disease	T	66				M
4. During meditation, our brains show alpha waves, a state of deep relaxation	T	69				M
5. Serotonin is a depression medication produced only in laboratories	F	95	*p* < 0.0020		*p* < 0.0001	M
6. The total number of neurons determines the power of our memory and general cognition	F	74			*p* < 0.0001	NM
7. Anxiety is caused by chemical disturbances in the brain	T	70	*p* < 0.0307			M
8. Every neuron stores different information	F	57			*p* < 0.0001	NM
9. We use our brains 24 h a day	T	93	*p* < 0.0326			NM
10. Magnetic Resonance Imaging can be used to see what people are thinking	F	94				M
11. There are critical or sensitive periods during childhood after which certain things become more difficult to learn, such as piano or languages	T	65	*p* < 0.0032			NM
12. Multiple Sclerosis can begin at any age	T	83		*p* < 0.0008		M
13. All stroke patients lose their speech	F	99		*p* < 0.0018	*p* < 0.0273	M
14. Drugs do not alter the brain’s biochemical composition, but they do alter behavior	F	74	*p* < 0.0027		*p* < 0.0109	M
15. Cell death in Parkinson’s disease causes motor symptoms such as freezing and tremors	T	90		*p* < 0.0142		M
16. Vaccines cause autism in developing children	F	99		*p* < 0.0129		M
7. Although we only remember small parts of our dreams, dreams are long and happen in “real time” relative to the events they represent	T	41		*p* < 0.0234		NM
18. Each region of the brain has a unique function	F	47	*p* < 0.0166		*p* < 0.0001	NM
19. Neuroplasticity, the nervous system’s ability to change and adapt, ends after adolescence	F	87			*p* < 0.0010	M
20. Humans are the only living beings with consciousness	F	55	*p* < 0.0094		*p* < 0.0099	NM
21. Our imagination can create false memories; events we believe we experienced but never happened	T	94		*p* < 0.0195	*p* < 0.0265	M
22. Larger brains are smarter	F	93				NM
23. The best prevention against Alzheimer’s disease is physical exercise	T	51			*p* < 0.0093	M
24. During sleep, our brain activity decreases	F	37			*p* < 0.0026	NM
25.IQ scores may change over time	T	84	*p* < 0.0001		*p* < 0.0003	NM
26. When we see different colors in a dress or sneakers, it is because we are using the dominant side of our brain (right vs. left)	F	39			*p* < 0.0023	NM
27. The period between 0 and 3 years of age is a very important period of neuronal growth and proliferation. For better performance in life, children must be exposed to all possible stimuli during this period, such as math, language and music	F	25	*p* < 0.0240			NM
28. Epilepsy is not contagious, but can be inherited	T	92				M
29. Using a tablet or cell phone during the first years of life can positively influence a child’s development	F	77		*p* < 0.0204	*p* < 0.0325	M
30. During hypnosis, we completely lose consciousness	F	73			*p* < 0.0001	NM

*The 30 survey statements listed in the order of presentation, which was the same for all participants. CA: correct answer (true or false); %: percent of patients who answered correctly; Age/Region/Prof: p-values for questions that differed based on Age, Region or Profession. SC: statement category (M: questions often covered in the media; NM: statements about classic neuromyths).*

## Results

### Score Distribution

We first tested and confirmed the normality of the score distribution (Anderson-Darling test, A2 = 6.4925, *p* < 0.0001; curve coefficients μ = 0.7059 ± 0.0028, σ = 0.0937 ± 0.0064). In addition, a Principal Components Analysis, which estimates correlations by the Row-wise method, revealed no correlation among our three independent variables (age, region, profession; see Supplementary Table 3).

### Multiple Regression

Next, to determine the effect of each of the variables of interest on participants’ performance, we conducted a multiple regression analysis including all variables of interest and their interactions, *F*(14,1113) = 9.0102, *p* < 0.0001. The test revealed a significant contribution of each of the variables, with the strongest contribution being that of Profession. Effect tests: Age (F ratio 3.0971, *p* = 0.0088); Region (F ratio 3.2083, *p* = 0.0070); Profession (F ratio 17.9602, *p* < 0.0001). None of the interactions reached significance (all *p*s > 0.05). For Age, *post hoc* Tukey HSD tests revealed significantly lower performance by the 60 and older group compared with the 20-29-year-old group (*p* = 0.0453) and the 30-39-year-old group (*p* = 0.0418), respectively. In terms of Region, respondents in the Southeast performed significantly better than those in the Northeast (*p* = 0.0315). Finally, in terms of Profession, the Biological sciences group answered significantly better than all other groups (Exact sciences: *p* = 0.0002; Humanities: *p* < 0.0001; Health: *p* = 0.0014; Other: *p* < 0.0001), and the Other/retired/not working group performed significantly worse than all other groups (Exact sciences: *p* = 0.0063; Humanities: *p* < 0.0001; Health: *p* < 0.0001).

### Analyses of Variance

**FIGURE 2:**
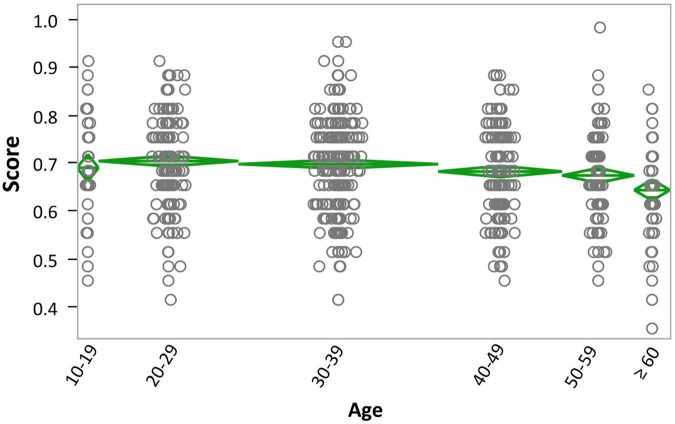
Age × overall score. Overall score for participants in the different age groups: 10–19 years old, 20–29 years old, 30–39 years old, 40–49 years old, 50–59 years old, and 60 or older. Data are shown in mean diamond graphs, where the width of the diamond is directly proportional to the sample size, and the height corresponds to the variance. No intersection between diamonds implies rejection of the null hypothesis for an α error of 5%. The circle markers represent each participant’s score.

To further assess effects within each variable, we conducted one-way Analyses of Variance. The main effect of Age was significant, *F*(5,1122) = 6.36, *p* < 0.0001, η_p_^2^ = 0.03, with participants in the 20–29 group responding best and individuals in the 60 and older group responding worst (Figure 2; see Supplementary Table 4 for means and SEMs). The Levene’s test for equality of variance was not significant (*p* = 0.4021), indicating homoscedasticity. Furthermore, *post hoc* Tukey HSD tests revealed that the 60+ group responded worse than the 20–29, the 30–39 and the 40–49 groups, respectively, and the 50-59 group responded worse than the 20–29 group (see Supplementary Table 5).

**FIGURE 3:**
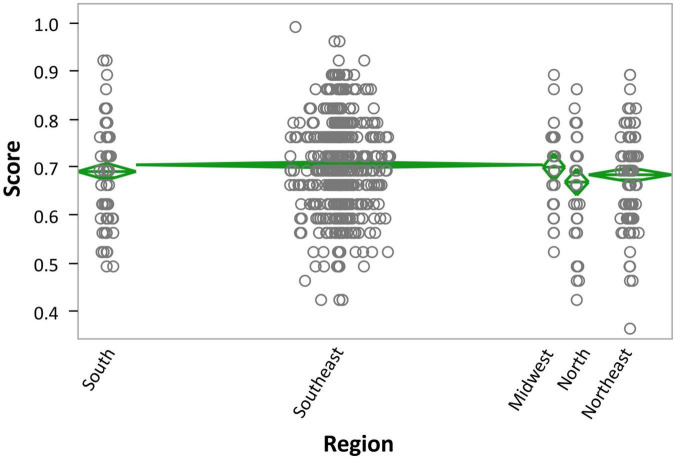
Region × Score. Data are shown in mean diamond graphs, where the width of the diamond is directly proportional to the sample size, and the height corresponds to the variance. No intersection between diamonds implies rejection of the null hypothesis for an α error of 5%. The circle markers represent each participant’s score.

To assess the effect of Region, we excluded participants who did not declare their region of origin (*n* = 24). For the remaining participants (*n* = 1,104), the main effect of Region was also significant, *F*(4,1099) = 3.10, *p* < 0.0150, η_p_^2^ = 0.01, with participants from the Southeast obtaining the highest scores and individuals from the North obtaining the lowest scores (see Supplementary Table 6). The Levene’s test for equality of variance was not significant (*p* = 0.3837), indicating homoscedasticity. No *post hoc* comparisons reached significance (see Figure 3).

**FIGURE 4:**
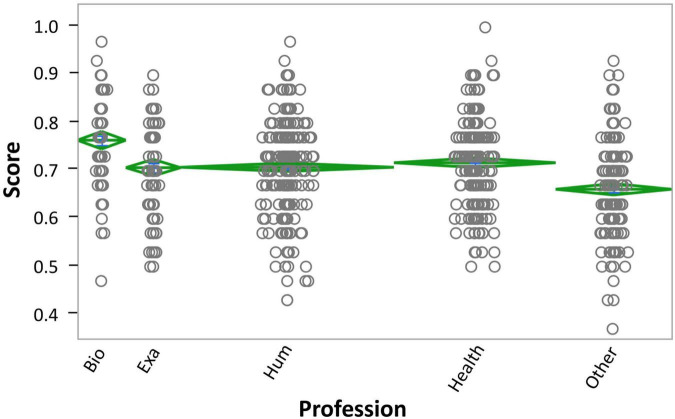
Profession × overall score. Individuals reported studying or working in the areas of Biological Sciences (Bio), Exact sciences (Exa), Humanities (Hum), Health, or Other (other, retired, or not working). Data are shown in mean diamond graphs, where the width of the diamond is directly proportional to the sample size, and the height corresponds to the variance. No intersection between diamonds implies rejection of the null hypothesis for an α error of 5%. The circle markers represent each participant’s score.

Finally, the main effect of Profession was also significant, *F*(4,1123) = 24.12, *p* < 0.0001, η_p_^2^ = 0.08, with participants who declared working in the biological sciences (Bio) responding best and individuals who declared ‘other, retired or not working’ (Other) responding worst (see Supplementary Table 7). The Levene’s test for equality of variance was not significant (*p* = 0.3617), indicating homoscedasticity. Furthermore, *post hoc* Tukey HSD tests revealed that the Bio group performed significantly better than all others, while people in the exact sciences (Exa), humanities (Hum) and Health groups differed only from the Other group and not from each other (see Figure 4 and Supplementary Table 8).

Next, we were interested in taking a closer look at effects within Region and Profession. First, we wanted to know whether Age made a difference in neuroscience-related knowledge in each of the six regions studied. This effect was significant in only three regions: Southeast, Midwest and Northeast.

In the Southeast, scores decreased from youngest to oldest [*F*(5,750) = 3.21, *p* = 0.007, η_p_^2^ = 0.02; Supplementary Table 9], and *post hoc* Tukey HSD tests revealed that the 60 + group differed significantly from the 20-29 to 30-39 groups, respectively (Supplementary Table 10).

Similarly, in the Midwest, age groups followed a similar pattern, *F*(4,36) = 3.79, *p* = 0.0113 η_p_^2^ = 0.30, with the 20–29 group performing best, followed by the 30–39 group, then the 50–59 and 40-49 groups, and finally the 60 + group (see Supplementary Table 11). *Post hoc* Tukey HSD tests revealed that the 60 + group differed significantly from the 20–29 to 30–39 groups, respectively (see Supplementary Table 12).

Finally, in the Northeast, 20–29 year-olds answered best, followed by 40–49 year-olds and 30–39 year-olds, then 10–19 year-olds, 50–59 year-olds, and finally the 60 + group, *F*(5,148) = 3.94, *p* = 0.0022, η_p_^2^ = 0.12 (Supplementary Table 13). *Post hoc* Tukey HSD tests revealed that the 60 + group performed significantly worse than the 20–29, 30–39, and 40–49 groups, respectively (Supplementary Table 14).

We then investigated whether profession made a difference in each of the regions, and effects were significant for three regions as well: South [*F*(4,104) = 2.90, *p* = 0.0255, η_p_^2^ = 0.10], Southeast [*F*(4,751) = 13.98, *p* < 0.0001, η_p_^2^ = 0.07], and Northeast [*F*(4,149) = 4.48, *p* = 0.0019, η_p_^2^ = 0.11]. Thus, in two regions (Midwest and North), profession did not seem to influence neuroscience-related knowledge.

As seen in the overall analyses, people in the Other category performed worst in all three regions where profession had an effect. Also in line with the overall analyses, people in the Biological sciences group performed best in the Southeast and Northeast, while the other professions did not differ from each other (see Supplementary Tables 15–18). Interestingly, in the South, the Exact sciences group performed best, followed by Health, then Humanities, then the Biological group, and finally the Other group. Only one *post hoc* Tukey HSD test was significant, revealing that the Other group performed worse than the Exact sciences group (*p* = 0.0439; see Supplementary Table 19).

When we asked whether Age made a difference in neuroscience-related knowledge in each of the professions studied, no effects reached significance. Finally, a Pearson’s multivariate regression analysis showed no significant correlation between participant performance and total years of schooling or total years in current profession.

### Media Coverage Versus Classic Neuromyths

As mentioned in the introduction, prior to our analyses we hypothesized that respondents would perform better on statements about topics often covered in the media (e.g., vaccines, autism, child development and neurodegenerative disorders such as Alzheimer’s and Parkinson’s diseases) than on statements about common neuromyths (e.g., brain size and IQ, male vs. female brains). Thus, we divided the 30 statements into the two categories (see Table 5) and conducted a Multivariate analysis of variance (MANOVA), *F*(76,1051) = 1.46, *p* = 0.0073, which confirmed our hypothesis: overall, individuals performed better on the Media statements (overall mean = 0.83) than on neuromyths (overall mean = 0.56). See Supplementary Tables 20–22 for means and SEMs for each of the variables.

In terms of age, *F*(5,1122) = 2.43, *p* = 0.0333, η_p_^2^ = 0.01, the 40–49 group showed the biggest discrepancy in performance between question categories, followed by the 60 + group, then the 50–59 group, the 30–39 group, 10–19 group and 20–29 group. The only significant *post hoc* Tukey HSD test showed that the 40–49 group differed significantly from the 20 to 29 group (*p* = 0.0215).

In terms of region, *F*(4,1099) = 2.59, *p* = 0.0354, η_p_^2^ = 0.01, the Northeast showed the biggest discrepancy, followed by the North, then the Southeast, the South and the Midwest. No *post hoc* comparisons reached significance.

Finally, in terms of profession, *F*(4,1123) = 6.45, *p* < 0.0001, η_p_^2^ = 0.01, the Other group showed the biggest discrepancy, followed by the Exact science group, then the Humanities, Health, and Biological sciences group. Three significant *post hoc* Tukey HSD tests showed that the Other group differed significantly from the Health (*p* = 0.0030), Humanities (*p* = 0.0020), and Biological sciences groups (*p* < 0.0001), respectively.

## Discussion

In order to test general knowledge about Neuroscience in a sample of Brazilian individuals from varied backgrounds, we created a 30-item questionnaire that aimed to cover a range of neuroscience topics as well as common neuromyths. Besides including questions previously asked by authors in other countries, we searched for the neuroscience-related questions that showed up most often in Google searches conducted in Brazil in Portuguese.

While participants overall had relatively good knowledge of some pathologies (e.g., stroke, epilepsy, Parkinson’s Disease and Multiple Sclerosis), the role of serotonin and the concept of neuroplasticity, most participants endorsed classic neuromyths (the period between 0 and 3, left- vs. right-hemisphere dominance, and using only 10% of the brain). Overall, the percent of correct responses ranged from 17 to 99%, and there were important differences in performance based on age, region and field of study or profession.

In terms of age, the second youngest group (20–29-year-olds) performed best overall, while participants in the oldest group (60 and older) responded worst. This difference may reflect the fact that information (neuroscience-related and otherwise) has recently permeated environments most commonly frequented by younger folks, such as colleges/universities, and especially online sources, including social media platforms (Chudler and Bergsman, 2014; Babinski et al., 2018; Falk et al., 2013). In Brazil, internet use is highest among 18–29-year-olds (90–91%), and lowest among individuals 60 and older (38.7%), with numbers steadily declining with increasing age (IBGE, 2018).

In terms of location within Brazil, people in the Southeast performed best, while people in the North responded worst. This finding is in line with what we know about inequalities across Brazilian regions in terms of access to education, internet and other resources, which generally favor the South and Southeast and are worst in the North and Northeast (IBGE, 2021). However, while the quality of education is mostly better in the more favored regions (and average number of years of education is higher), we know that nowadays, much information is accessed online. Further, while internet access does vary across regions (IBGE, 2021), we know that internet use is high and widespread in Brazil (We are Social, 2020). Thus, if the quality of information accessed (online courses or websites with medical or scientific information) is generally good in quality, the internet should improve access to information for people with lower education levels, potentially narrowing the gap between groups. In this study we question the quality of online science information because of the number of non-science online portals in Brazil that publish this type of information (IGC, 2018) as well as the exponential growth of science course offerings in recent years that are not associated with well-established higher learning institutes (see Introduction). Alternatively, our data may reflect the fact that better education gives people the tools they need to filter online information properly and access better quality sources while ignoring others. Future studies should investigate these ideas in further detail.

Finally, in terms of profession, individuals who declared working in the biological sciences most often answered best (in 4 out of 5 regions) (Drummond and Fischhoff, 2017), consistently ahead of individuals in the exact sciences, humanities, health sciences, and those who declared ‘other, not working or retired’. Surprisingly, individuals in the health sciences consistently answered below the biological sciences group and also did not differ significantly in performance from the exact sciences or humanities groups (Roffman, 2006; Gould et al., 2014; Goldenberg and Krystal, 2017). When we analyzed questions individually, the health group answered best on only one question (14: *Drugs do not alter the brain’s biochemical composition, but they do alter behavior -* correct answer: FALSE) This finding was surprising, as we hypothesized that people in the health sciences would perform similarly to people in the biological sciences and did not expect their performance to be similar to that of people in the exact sciences and humanities (e.g., accountants and lawyers).

Interestingly, in two regions (North and Midwest), profession did not influence knowledge in neuroscience (i.e., people in the biological and health sciences knew just as much – or little – as people in the exact sciences, humanities, or other areas). Additionally, in the South, people in the exact sciences outperformed everyone else, and people in the biological sciences in that region performed in second-to-last place (see Supplementary Table 19). Thus, while people in the sciences performed better overall, this was not true across all regions. This finding suggests that education and training in different fields may differ across regions and Brazil. This is another interesting area for future research.

Overall, six of the nine questions that participants answered best (90% correct or higher) had to do with pathologies, disorders or treatments (Q13 – Stroke, 16 – Vaccines, Q5 – Serotonin and Depression, Q10 – Magnetic Resonance Imaging, Q28 – Epilepsy, and Q15 – Parkinson’s Disease; see Table 1), while five of the seven with the lowest scores (50% correct or worse) had to do with brain anatomy, overall function or development (Q18 – brain regions, Q1 – we use 10% of our brain, Q26 – hemispheric dominance, Q27 – critical periods and Q2 – male vs. female brains). One possible explanation for the discrepancy between types of knowledge is that it may be easier to access valid sources of information regarding health issues, while anatomy and physiology are most often learned in directed forms of study (i.e., courses; see also Betts et al., 2019). Furthermore, misrepresentations about anatomy and physiology may easily appear in entertainment media (e.g., series and films that talk about the use of 10% of the brain, online news that test whether people see different colors on a dress or sneakers and claim the answer depends on hemispheric dominance) (Brainard and Hurlbert, 2015; Gegenfurtner et al., 2015; Lafer-Sousa et al., 2015; Michel, 2015; Feitosa-Santana et al., 2018).

It is interesting to observe that while most people knew that larger brains do not mean smarter brains (Q22), other classic neuromyths were still prevalent (Q1, Q26 and Q2; see Table 5). And while most correctly answered that we use our brains 24 h a day (Q9), most incorrectly responded that brain activity decreases during sleep (Q24). The notion that larger brains are smarter (Q22) may be considered an “older” myth that people may have become familiar with when studies regarding brain evolution and the encephalization quotient first appeared a few decades ago (Deaner et al., 2007). Because these notions are somewhat older, over the years laypeople may have been exposed to more updated information that may have appeared in the media as curiosities (e.g., it’s not the size or number of neurons that matter, but our synapses or connections; or, Einstein’s brain was not that large after all! Salvatori, 1999; Falk, 2009; Hines, 2014). On the other hand, the notion that some people use one hemisphere more than the other (i.e., some people are predominantly “left- or right-brained”) may have been reinforced more recently by viral phenomena such as “The Dress” (Lafer-Sousa et al., 2015).

When we divided our statements into those most often covered in the media versus more common neuromyths (see Table 5), we discovered that performance was significantly better on the media statements across groups, as predicted. While information can often be misconstrued in the media, it seems like some correct information does get through; furthermore, the media may also reinforce some neuromyths, as discussed above. Overall, the data suggest that neuromyths continue to be hard to eliminate across people from different walks of life.

Perhaps one of the most controversial issues tested was question 16: *Vaccines cause autism in developing children* (correct answer: FALSE). This neuromyth has invaded the media and taken hold of communities from all cultural and socioeconomic levels worldwide, with serious global health consequences (anti-vaccination movements, etc.) (Chiou and Tucker, 2018; Lavorgna et al., 2018). Great efforts have been made to tear this myth down, and we were pleasantly surprised to see that overall, it was the second ranked question, with participants answering with 99% accuracy. However, this question was influenced by region, with the North answering worst, suggesting there is still some work to be done in regions where quality education is not as readily available.

Another timely issue was presented in question 29 *– Using* a tablet or cell phone during the first years of life can positively influence a child’s development (correct answer: FALSE). Interestingly, this was the only question where people in the humanities group answered best, followed by the Bio and health groups, and then the Other and exact groups. It is unclear why the humanities group would answer best, but since it is a timely topic that affects anyone caring for children, it follows that people from all areas would be interested in learning more about this issue.

To make sure the differences among groups did not reflect other variables intrinsic to the groups sampled, we conducted a Pearson’s multivariate regression analysis, which showed no significant correlation between participant performance and total years of schooling or total years in current profession. Given that overall knowledge in neuroscience was generally low and that several respondents declared having completed several graduate-level courses (18+ years of education), this likely suggests that graduate programs in the neurosciences or related areas are limited in quality and/or effectiveness. Indeed, more years of study or work do not guarantee greater knowledge in neuroscience, even for those in the areas of health or biological sciences. Furthermore, the finding that Age did not influence Profession corroborated this finding: within each profession, chronological age (which should be strongly correlated with years of education and experience) did not influence neuroscience-related knowledge.

While one of our aims was to include questions identified as having raised the interest of the target population, a few questions may have been particularly difficult, given the nature of the topic or the way in which they ere worded. We noticed this only *a posteriori*, and the fact that most groups answered below chance on those questions supports our assumptions and precludes us from making any observations regarding group effects. The first of these was question 2: *Structural differences between male and female brains are so obvious that any professional can identify a person’s gender simply by looking at an image of their brain (correct answer: FALSE).* Structural and functional brain differences between the sexes have been reported in several academic publications, albeit in the context of group effects that considered large groups of participants. More recently, studies have argued that intragroup differences (women vs. other women or men vs. other men) are larger than intergroup ones (men vs. women), suggesting female or male structural characteristics cannot be identified on individual brain scans (Joel, 2011; Ingalhalikar et al., 2014). This last piece of information, however, which includes knowledge about the expertise of neuroimaging professionals, is likely beyond the scope of knowledge of people who are not specialists in this area or who have not done research specifically within this topic. Thus, answering this question incorrectly may not be a fair indicator of the quality of higher education or freely available sources of neuroscientific information.

Similarly, most groups answered below chance on question 17: *Although we only remember small parts of our dreams, dreams are long and happen in “real time” relative to the events they represent* (correct answer: TRUE). The original idea with this question was to tear down the myth that dreams represent signs of the divine, insights of future events, or symbolic clues from other worlds. A correct interpretation is that dreams are one way our brains process acquired information and crystalize memories (Wamsley, 2014). However, since the area of dreams is a very specific area of research that most people (even those in the health or science areas) may not have contact with, question 17 may also not be very informative.

Finally, we also had doubts about question 27: *The period between 0 and 3 years of age is a very important period of neuronal growth and proliferation. For better performance in life, children must be exposed to all possible stimuli during this period, such as math, language and music (correct answer: FALSE).* The wording in this question may have led to errors, as it may contain a “catch”: the question speaks specifically of stimuli such as math, language and music – which would not result in any measurable benefit during these early years, considering children have not completely developed more basic functions such as vision, audition and motor skills. However, this may be confusing, since it is undeniable that various types of stimuli during this period are positive and necessary for normal development. While we tried to emphasize the insignificant (or even negative effect) excessive stimulation could have during this period, in retrospect, the question may not have adequately captured this idea. Thus, participants may have responded incorrectly based on some correct knowledge (i.e., that age-appropriate stimulation during the first three years of life can have a positive effect on development).

While yielding relatively high overall scores, two additional statements may raise questions: 10 (*Magnetic Resonance Imaging can be used to see what people are thinking*), and 11 (*There are critical or sensitive periods during childhood after which certain things become more difficult to learn, such as piano or languages*). A total of 94% of respondents indicated that statement 10 is False (which was the answer we intended to elicit). While MRI technology can reveal a lot about relative engagement of different brain regions on specific tasks of interest, it is not a method that allows us to read complex thoughts verbatim (as sometimes depicted in films or series) or even determine indirect mental states (e.g., whether someone is guilty, as in the proposed use of fMRI in a court of law). Thus, our goal with that statement was to assess whether individuals knew the relative limitations of that technology. For statement 11, 65% of respondents indicated it was true (our intended answer). While it may have been better to use only the term “sensitive,” the term “critical” is older and probably more well-known, which is why we chose to keep the statement in that form (“critical or sensitive”). And, while controversy exists regarding how determinant such periods are for learning specific skills, little doubt exists in the scientific community that neuroplasticity gradually decreases and that this is likely linked to sensitive periods.

It is important to note that our study design requires that people be literate and have access to the internet. Thus, while we obtained responses from a large sample of Brazilians from all five geographical regions, age groups and several different professions, our sample does not represent the 11 million Brazilians over the age of 15 who are illiterate (EBC, 2020). Also, internet use is not the same across regions: a study from 2018 by the Brazilian Institute for Geography and Statistics (IBGE) found interregional differences in internet use (81.1% and 78.2% of people living in the Southeast and South use the internet, compared with 64.7% and 64% of people in the North and Northeast, respectively) (Markram, 2013). Our study also requires respondents to be interested in the topic and be motivated to respond, as all answers were voluntary. Furthermore, the method of data collection used in this study is known as snowball sampling, meaning we sent the survey to our contacts, who in turn shared it with their own contacts. While this type of sampling has the advantage of increasing reach (i.e., participants are more likely to respond when invited by people they know), this could create a non-random sample that may not perfectly generalize to the population at large. Thus, while online surveys have the advantage of quickly reaching many people in different locations, they are limited by the considerations listed above. While a design targeting specific populations (including people less interested in the topic who may not participate voluntarily) could reach individuals not included in the current survey, such designs carry additional methodological constraints (e.g., how to interpret responses from people who felt pressured to respond, such as in a classroom?). Future studies should explore how to obtain a more random sample while avoiding these additional experimental limitations.

## Conclusion

Access to quality information and accurate knowledge about how the brain and nervous system work are essential parts of constructing a better-informed society. Such access can also help people better take care of their own health, as well as become better professionals, particularly in the areas of health (e.g., nurses and doctors), biological sciences, or even education.

A growing interest in Neuroscience-related knowledge in recent years has led to an exponential growth in the amount of related information (correct or not) made available online as well as the market for Neuroscience-related courses in Brazil. Despite this growing interest and course availability, Brazilians from all walks of life show poor knowledge in this field. We observed this even among people studying or working in the areas of biological or health sciences, and even among those reporting several years of graduate education or professional experience, suggesting much work needs to be done to improve the quality of (neuro)science-related course options. While overall, participants seemed to know more about themes that are often presented in the media, they all displayed high endorsement of common neuromyths (e.g., left- vs. right-hemisphere dominance, and using only 10% of the brain). We also observed differences among Brazilian regions, which reflect long-standing inequalities in terms of access to quality education and other resources. Thus, professionals seeking to improve the quality of scientific content and communication (in courses or otherwise) may begin by focusing on ways of combatting neuromyths and developing ways of reaching individuals in the health sector as well as those living in disadvantaged regions. To the best of our knowledge, this is the first study testing these questions in such a large sample of Brazilians from all regions and several walks of life. We hope future studies further explore these questions and others that were raised here and remain unanswered.

## Data Availability Statement

The raw data supporting the conclusions of this article will be made available by the authors, without undue reservation.

## Ethics Statement

Ethical review and approval was not required for the study on human participants in accordance with the local legislation and institutional requirements. The patients/participants provided their written informed consent to participate in this study.

## Author Contributions

FP and ES conducted the word searches and created the survey. AA conducted the statistical analyses. FP, ES, and AA drafted the manuscript. All authors distributed the survey among their personal and professional contacts, reviewed and discussed the findings, and reviewed and approved the final manuscript.

## Conflict of Interest

The authors declare that the research was conducted in the absence of any commercial or financial relationships that could be construed as a potential conflict of interest.

## Publisher’s Note

All claims expressed in this article are solely those of the authors and do not necessarily represent those of their affiliated organizations, or those of the publisher, the editors and the reviewers. Any product that may be evaluated in this article, or claim that may be made by its manufacturer, is not guaranteed or endorsed by the publisher.

## Footnotes

1
https://www.saude.gov.br/fakenews
