# Supplementary material for: What Does the General Public Know (or Not) About Neuroscience? Effects of Age, Region and Profession in Brazil
Source: Front Hum Neurosci. 2022 Mar 4;16:798967. doi: 10.3389/fnhum.2022.798967 (PMC8930840; doi:10.3389/fnhum.2022.798967)
Supplement: Supplementary file 1 [file Data_Sheet_1.pdf]

Supplementary materials

Supplementary Table 1. Google Adwords Report 05/06/2019

Previous research

Most clicked keywords

Keyword - Portuguese	Keyword - English	Estimated Clicks	Estimated Impressions
**Depressão**	**Depression**	**26,29**	**551,47**
**Ansiedade**	**Anxiety**	**14,53**	**323,00**
**Enxaqueca**	**Migraine**	**12,77**	**248,99**
**Fibromialgia**	**Fibromyalgia**	**7,24**	**161,08**
**Ressonância Magnética**	**MRI**	**6,83**	**161,33**
**Autismo**	**Autism**	**6,68**	**171,21**
**Memória**	**Memory**	**5,45**	**131,78**
**Alzheimer**	**Alzheimer's**	**5,05**	**126,10**
**Meditação**	**Meditation**	**4,49**	**129,18**
**Hipnose**	**Hypnosis**	**2,47**	**78,98**
**inteligencia artificial**	**artificial intelligence**	**1,51**	**65,00**
**Parkinson**	**Parkinson**	**1,37**	**36,19**
**Neuropsicologia**	**Neuropsychology**	**1,13**	**35,13**
**transtorno bipolar**	**bipolar disorder**	**1,11**	**25,28**
**AVC**	**Stroke**	**1,02**	**22,08**
**Esquizofrenia**	**Schizophrenia**	**929,00**	**26,46**
**Sonhos**	**Dreams**	**923,00**	**13,98**
**Células-tronco**	**Stem cells**	**881,00**	**18,36**
**Eletroencefalograma**	**Electroencephalogram**	**825,00**	**22,89**
**bulimia**	**bulimia**	**809,00**	**36,23**
**Aneurisma**	**Aneurysm**	**808,00**	**23,28**
**Epilepsia**	**Epilepsy**	**800,00**	**20,81**
**GABA**	**GABA**	**789,00**	**31,55**
**Esclerose Múltipla**	**Multiple sclerosis**	**744,00**	**14,63**
**Serotonina**	**Serotonin**	**690,00**	**15,78**
**Doppler**	**Doppler**	**690,00**	**17,98**
**TDAH**	**ADHD**	**644,00**	**17,15**
**Hormônios**	**Hormones**	**633,00**	**13,64**
**Síndrome do Pânico**	**Panic Syndrome**	**576,00**	**13,12**
**cafeina**	**caffeine**	**517,00**	**16,85**
Dislexia	Dyslexia	470,00	10,07
Paralisia cerebral	Cerebral palsy	457,00	16,18
Glutamato	Glutamate	431,00	11,73

Meningite	Meningitis	401,00	10,01
Tumor cerebral	Brain tumour	367,00	10,02
Acidente Vascular Cerebral	Stroke	355,00	9,69
Apraxia	Apraxia	303,00	3,22
transtorno de atencao	attention disorder	294,00	4,77
Paralisia de Bell	Bell's palsy	266,00	9,88
Melatonina	Melatonin	236,00	5,45
Epinefrina	Epinephrine	204,00	4,01
Oxitocina	Oxytocin	197,00	6,61
TMS	TMS	193,00	8,05
Aneurisma cerebral	Brain aneurysm	185,00	6,46
transtorno de borderline	borderline disorder	178,00	3,52
Neuromarketing	Neuromarketing	171,00	8,50
Fadiga	Fatigue	163,00	4,02
Consciência	Consciousness	151,00	3,74
Tremor essencial	Essential tremor	114,00	2,73
TOC	TOC	113,00	3,87
angiografia	angiography	87,00	2,32
transtorno obsessivo compulsivo	obsessive-compulsive disorder	81,00	1,91
Dopamina	Dopamine	75,00	1,77
Música clássica	Classic music	72,00	1,17
Asperger	Asperger	69,00	1,73
Demência	Insanity	66,00	2,28
Esclerose lateral amiotrófica	Amyotrophic lateral sclerosis	57,00	1,30
Polineuropatia	Polyneuropathy	54,00	1,20
ensino crianças	teaching children	52,00	1,18
Tomada de decisão	Decision-making	49,00	1,32
anorexia nervosa	anorexia nervosa	45,00	990,00
MD	MD	44,00	1,02
programacao neurolinguistica	neurolinguistic programming	44,00	1,95
coma	with the	42,00	990,00
Cognição	Cognition	42,00	1,34
Transtorno de Ansiedade Generalizada	Generalized Anxiety Disorder	40,00	919,00
Convulsão	Seizure	36,00	739,00
Discalculia	Discalculia	35,00	667,00
alexia	alexia	35,00	831,00
transtorno desafiador opositor	opposing challenging disorder	34,00	340,00
Sabores	Flavors	34,00	797,00
tomografia computarizada	computed tomography	30,00	646,00
Detector de mentiras	Lies detector	29,00	424,00
Aspartato	Aspartate	28,00	550,00
Emoções	Emotions	25,00	894,00
transtorno de personalidade	personality disorder	24,00	583,00
Espectro Autista	Autistic Spectrum	22,00	457,00

Neuroplasticidade	Neuroplasticity	22,00	768,00
sonambulismo	sleep-walking	21,00	365,00
Brain training	Brain training	21,00	457,00
Miopatia	Myopathy	20,00	525,00
Huntington	Huntington	20,00	369,00
Distonia	Dystonia	19,00	865,00
Neuroanatomia	Neuroanatomy	19,00	495,00
IQ scores	IQ scores	19,00	457,00
ataxia	ataxia	17,00	1,05
Giros	Rotations	17,00	302,00
acetilcolina	acetylcholine	14,00	185,00
Encefalopatia	Encephalopathy	13,00	210,00
Afasia	Aphasia	12,00	147,00
Propriocepção	Proprioception	11,00	365,00
Psicopatia	Psychopathy	11,00	122,00
Síndrome da Fadiga Crônica	Chronic Fatigue Syndrome	11,00	151,00
Espasticidade	Spasticity	11,00	432,00
Hipocampo	Hippocampus	9,00	88,00
Cinetose	Ketosis	8,00	59,00
Peptídeos	Peptides	8,00	118,00
Cisto aracnoide	Arachnoid cyst	7,00	59,00
Parkinson-plus	Parkinson-plus	7,00	59,00
L-Dopa	L-Dopa	7,00	332,00
neurogenesis	neurogenesis	7,00	92,00
Cíngulo	Cingulate	7,00	180,00
Sistema Nervoso Periférico	Peripheral Nervous System	6,00	88,00
Cefaleia em salvas	Cluster headache	6,00	122,00
sindrome de Williams	Williams syndrome	5,00	29,00
Redes Neurais Artificiais	Artificial neural networks	5,00	210,00
psicopatia	psychopathy	5,00	147,00
Psicossomático	Psychosomatic	4,00	147,00
Glicina	Glycine	4,00	92,00
Encefalite	Encephalitis	4,00	122,00
ansiolitico	anxiolytic	4,00	122,00
Encéfalo	Brain	4,00	88,00
Síndrome de Turner	Turner syndrome	4,00	92,00
Demência com corpos de Lewy	Dementia with Lewy bodies	4,00	29,00
estimulacao transcraniana	transcranial stimulation	3,00	59,00
Tetraplegia	Tetraplegia	3,00	88,00
Corpo Caloso	Callous Body	3,00	63,00
Nervo espinhal	Spinal nerve	3,00	29,00
Sistema Nervoso Central	Central Nervous System	3,00	59,00
Trauma raquimedular	Spinal trauma	3,00	155,00
antidepressivo	antidepressant	3,00	122,00
transtorno dissociativo	dissociative disorder	3,00	92,00
Paralisia supranuclear progressiva	Progressive supranuclear palsy	3,00	29,00
Mioclonia	Myoclonia	3,00	29,00
REM	REM	3,00	59,00

Lobo frontal	Frontal lobe		3,00	29,00
transtorno de estresse pós-traumático	post-traumatic stress disorder		2,00	59,00
Medula espinhal	Spinal cord		2,00	29,00
Sistema Límbico	Limbic System		2,00	88,00
Decorticação	Decortication		2,00	29,00
Distonia focal	Focal dystonia		2,00	88,00
Oliver sacks	Oliver sacks		2,00	59,00
Mielite transversa	Transverse myelitis		1,00	29,00
Neurogênese	Neurogenesis		1,00	63,00
Nervo alveolar inferior	Lower alveolar nerve		1,00	29,00
Eric Kandel	Eric Kandel		1,00	29,00
Psicopata	Psycho		1,00	29,00
Epilepsia abdominal	Abdominal epilepsy		0,00	29,00
10 porcento do cérebro	10 percent brain	-	-	
Abarognose	Abarognosis	-	-	
Abulia	Abulia	-	-	
Acalculia	Acalculia	-	-	
Acefalia	Acefalia	-	-	
Ácido Serina	Serine Acid	-	-	
adicao	addition	-	-	
Afasia de Wernicke	Wernicke's aphasia	-	-	
Agenesia de corpo caloso	Agenesis of corpus callosum	-	-	
Agnosia	Agnosia	-	-	
agrafia	agraphia	-	-	
Amígdala cerebral	Cerebral amygdala	-	-	
Amusia	Amusia	-	-	
Andreas Versalius	Andreas Versalius	-	-	
Aneurisma de Charcot-Bouchard	Charcot-Bouchard aneurysm	-	-	
anfetamina	amphetamine	-	-	
anomia	anomie	-	-	
anosmia	anosmia	-	-	
Anosognosia	Anosognosia	-	-	
Aracnóide	Arachnoid	-	-	
Area de Broca	Drill Area	-	-	
Área de Wernicke	Wernicke area	-	-	
Ataque paroxístico	Paroxysmal attack	-	-	
Axônio	Axon	-	-	
Calcitonina	Calcitonin	-	-	
canabis	cannabis	-	-	
Catalepsia	Catalepsy	-	-	
Catalepsia patológica	Pathological catalepsy	-	-	
CDKL5	CDKL5	-	-	
Cerebelo	Cerebellum	-	-	
cerebro direito	right brain	-	-	
cerebro esquerdo	left brain	-	-	
Cérebro feminino	Female brain	-	-	
Cérebro masculino	Male brain	-	-	
Choreia	Cry	-	-	

cocaina	cocaine	-	-
Colpocefalia	Colpocephaly	-	-
Convulsão febril	Febrile seizure	-	-
Coreia de Sydenham	Sydenham Korea	-	-
Coreia gravídica	Pregnant korea	-	-
Corpos Mamilares	Nipple Bodies	-	-
Córtex Motor	Cortex Motor	-	-
Crise oculogírica	Oculogyric crisis	-	-
Degeneração Walleriana	Wallerian degeneration	-	-
Dentrito	Dentrito	-	-
Desordens do espectro alcoólico fetal	Fetal alcohol spectrum disorders	-	-
Diencéfalo	Diencephalon	-	-
Disfrenia tardia	Late dysphrenia	-	-
Disfunção cerebral	Cerebral dysfunction	-	-
Disfunções cerebrais	Brain dysfunctions	-	-
Dismetria	Dysmetria	-	-
Disosmia	Dysosmia	-	-
Dissecção da artéria vertebral	Vertebral artery dissection	-	-
Distrofia simpático-reflexa	Reflex sympathetic dystrophy	-	-
Doença de Alexander	Alexander's disease	-	-
Doença de Creutzfeldt-Jakob	Creutzfeldt-Jakob disease	-	-
Doença de Huntington	Huntington's disease	-	-
Doença de Niemann-Pick	Niemann-Pick disease	-	-
Doença de Pick	Pick's disease	-	-
Doença de Tay-Sachs	Tay-Sachs disease	-	-
Doença de Wilson	Wilson's disease	-	-
Doença do neurônio motor	Motor neuron disease	-	-
Doença infecciosa do sistema nervoso	Infectious disease of the nervous system	-	-
Dura mater	Dura mater	-	-
Encefalite letárgica	Lethargic encephalitis	-	-
Encefalomielite	Encephalomyelitis	-	-
Encefalomielite autoimune experimental	Experimental autoimmune encephalomyelitis	-	-
Encefalopatia de Wernicke	Wernicke's encephalopathy	-	-
Encefalopatia espongiforme bovina	Bovine spongiform encephalopathy	-	-
Encefalopatia hipertensiva	Hypertensive encephalopathy	-	-
Ependimoma	Ependymoma	-	-
Esclerose difusa	Diffuse sclerosis	-	-
Esquizencefalia	Schizencephaly	-	-
Estado vegetativo	vegetative state	-	-
Fórnix	Fornix	-	-
Frenologia	Phrenology	-	-
Gama-aminobutírico	Gamma-aminobutyric	-	-
Glucagon	Glucagon	-	-
haxixe	hashish	-	-
heroína	heroin	-	-
Hidroencefalia	Hydroencephaly	-	-

Hipotálamo	Hypothalamus	-	-
inibidores seletivos da recaptação da serotonina	selective serotonin reuptake inhibitors	-	-
Ínsula	Insula	-	-
ISRS	SSRI	-	-
Karl Wernicke	Karl Wernicke	-	-
lesão da medula espinal	spinal cord injury	-	-
Lobo occipital	Occipital lobe	-	-
Lobo parietal	Parietal lobe	-	-
Lobo temporal	Temporal lobe	-	-
LSD	LSD	-	-
maconha	marijuana	-	-
Malformações cefálicas congênitas	Congenital cephalic malformations	-	-
Manganismo	Manganism	-	-
MDMA	MDMA	-	-
Medula oblonga	Oblong cord	-	-
membro fantasma	ghost member	-	-
memorias falsas	false memories	-	-
Meninge	Meninge	-	-
Mielina	Myelin	-	-
Mononeuropatia	Mononeuropathy	-	-
morte do cérebro	brain death	-	-
Nervo cutâneo medial do antebraço	Medial cutaneous nerve of the forearm	-	-
Nervo cutâneo medial do braço	Medial cutaneous nerve of the arm	-	-
Nervo motor	Motor nerve	-	-
Nervo olfatório	Olfactory nerve	-	-
Nervo suboccipital	Suboccipital nerve	-	-
Nervos intercostais	Intercostal nerves	-	-
Neuroma de amputação	Amputation neuroma	-	-
Neurónio	Neuron	-	-
Neurônio cinórrodo	Cynorodal neuron	-	-
Neurônio espelho	Mirror neuron	-	-
Neurônio motor	Motor neuron	-	-
Neurônios	Neurons	-	-
Neurorreceptor	Neuroreceptor	-	-
Neurotransmissor	Neurotransmitter	-	-
Nocicepção	Nociception	-	-
Norepinefrina	Norepinephrine	-	-
Núcleo Accumbens	Accumbens Core	-	-
Núcleo Caudado	Caudate Core	-	-
Paralisia bulbar	Bulbar palsy	-	-
Paralisia de Erb	Erb's palsy	-	-
Paralisia flácida	Flaccid paralysis	-	-
Paraparesia espástica familiar	Family spastic paraparesis	-	-
Paul Broca	Paul Broca	-	-
periodo critico	critical period	-	-
Perturbação do neurodesenvolvimento	Neurodevelopmental disorder	-	-

Perturbações do movimento	Movement disorders	-	-
Pia mater	Pia mater	-	-
poda neural	neural pruning	-	-
Polineuropatia amiloidótica familiar	Familial amyloidotic polyneuropathy	-	-
Postura anormal	Abnormal posture	-	-
potencial de acao	action potential	-	-
Potencial sináptico	Synaptic potential	-	-
Privação de sono	Sleep deprivation	-	-
Prosopagnosia	Prosopagnosia	-	-
Proteopatia	Proteopathy	-	-
Psicossomatização	Psychosomatization	-	-
Putamen	Putamen	-	-
Rapid Eyes Moviment	Rapid Eyes Moviment	-	-
receptor	receiver	-	-
Ressonância Magnética Funcional	Functional Magnetic Resonance	-	-
Santiago Ramón y Cajal	Santiago Ramón y Cajal	-	-
Simultanagnosia	Simultanagnosia	-	-
Sinapse	Synapse	-	-
Síndrome clínica isolada	Isolated clinical syndrome	-	-
Síndrome complexa de dor regional	Complex regional pain syndrome	-	-
Síndrome da mão alheia	Alien hand syndrome	-	-
Síndrome de abstinência alcoólica	Alcohol withdrawal syndrome	-	-
Síndrome de adaptação ao espaço	Space adaptation syndrome	-	-
Síndrome de Aicardi	Aicardi syndrome	-	-
Síndrome de Alpers	Alpers syndrome	-	-
Síndrome de Anton-Babinski	Anton-Babinski syndrome	-	-
Síndrome de Cockayne	Cockayne syndrome	-	-
Síndrome de Cotard	Cotard's syndrome	-	-
Síndrome de Duane	Duane syndrome	-	-
Síndrome de fasciculação benigna	Benign fasciculation syndrome	-	-
Síndrome de Fields	Fields syndrome	-	-
Síndrome de Fregoli	Fregoli syndrome	-	-
Síndrome de Gerstmann	Gerstmann syndrome	-	-
Síndrome de Kinsbourne	Kinsbourne syndrome	-	-
Síndrome de Leigh	Leigh syndrome	-	-
Síndrome de Melkersson-Rosenthal	Melkersson-Rosenthal syndrome	-	-
Síndrome de Miller-Dieker	Miller-Dieker syndrome	-	-
Síndrome de Phelan-McDermid	Phelan-McDermid syndrome	-	-
Síndrome de Rasmussen	Rasmussen syndrome	-	-
Síndrome de Rett	Rett syndrome	-	-
Síndrome de Reye	Reye's syndrome	-	-
Síndrome de Sturge-Weber	Sturge-Weber syndrome	-	-
Síndrome de Timothy	Timothy syndrome	-	-
Síndrome de Weber	Weber's syndrome	-	-
Síndrome pós-pólio	Post-polio syndrome	-	-

Sinestesia	Synesthesia	-	-
Sistema vestibular	Vestibular system	-	-
sociopatia	sociopathy	-	-
Somático	Somatic	-	-
Somatização	Summing	-	-
Somestesia	Somesthesia	-	-
split brain	split brain	-	-
Sulcos	Grooves	-	-
Tálamo	Thalamus	-	-
Taupatia	Taupathy	-	-
transtorno do deficit de atencao	attention deficit disorder	-	-
transtorno do deficit de atencao com hiperatividade	attention deficit hyperactivity disorder	-	-
vacinas causam autismo	vaccines cause autism	-	-
Vasopressina	Vasopressin	-	-
Vesícula sináptica	Synaptic vesicle	-	-
vicio em alcool	alcohol addiction	-	-
vicio em drogas	drug addiction	-	-

Supplementary Table 2. Sentences related to each keyword – simple Google Search

**Depressão**	**Depression**
depressão é doença	Is depression a disease
depressão teste	depression test
depressão causas	depression causes
tratamentos para depressão	depression treatments
depressão como ajudar	depression how to help
depressão e ansiedade	depression and anxiety
depressão sintomas físicos	physical symptoms depression
tipos de depressão	types of depression
**Ansiedade**	**Anxiety**
ansiedade teste	test anxiety
ansiedade e depressão	anxiety and depression
ansiedade sintomas 100	anxiety symptoms 100
crise de ansiedade como controlar	anxiety crisis how to control
sintomas de ansiedade e stress	anxiety and stress symptoms
ansiedade frases	anxiety phrases
ansiedade noturna sintomas	night anxiety symptoms
tipos de ansiedade	types of anxiety
**Enxaqueca**	**Migraine**
enxaqueca como aliviar	migraine how to relieve
enxaqueca causas	migraine causes
enxaqueca remedio	migraine remedy
enxaqueca alimentação	migraine food
enxaqueca onde doi	migraine where it hurts

enxaqueca sintomas	migraine symptoms
enxaqueca com aura	migraine with aura
tipos de enxaqueca	migraine types
**Fibromialgia**	**Fibromyalgia**
complicações da fibromialgia	fibromyalgia complications
fibromialgia pontos	fibromyalgia points
fibromialgia aposenta	fibromyalgia retires
fibromialgia pode matar	fibromyalgia can kill
fibromialgia cid	fibromyalgia cid
fibromialgia alimentação	fibromyalgia food
fibromialgia pdf	fibromyalgia pdf
fibromialgia diagnostico	diagnostic fibromyalgia
**Ressonância Magnética**	**MRI**
ressonancia magnetica preço	magnetic resonance Price
ressonancia magnetica como funciona	magnetic resonance how it works
ressonancia magnetica cranio	magnetic resonance skull
ressonancia magnetica preço popular	popular price magnetic resonance
ressonancia magnetica com contraste	magnetic resonance with contrast
ressonancia magnetica da coluna	magnetic resonance of the spine
ressonancia magnetica cabeça	magnetic resonance head
ressonancia magnetica joelho	knee magnetic resonance
**Autismo**	**Autism**
autismo causas	autism causes
sintomas de autismo	autism symptoms
autismo é doença	autism is disease
tipos de autismo	types of autism
o que é autismo infantil	what is infantile autism
autismo wikipedia	autism wikipedia
autismo frases	autism phrases
tratamentos para autismo	autism treatments
**Memória**	**Memory**
memória psicologia	psychology memory
tipos de memória	memory types
memória de procedimento	procedure memory
tipos de memória neuropsicologia	neuropsychology memory types
memória declarativa	declarative memory
memória explícita	explicit memory
memoria conceito	memory concept
tipos de memória humana	human memory types
**Alzheimer**	**Alzheimer's**
alzheimer causas	alzheimer's causes

alzheimer tratamento	alzheimer treatment
alzheimer sintomas	alzheimer's symptoms
alzheimer fases	alzheimer's stages
alzheimer wikipedia	alzheimer wikipedia
alzheimer diagnostico	alzheimer's diagnosis
alzheimer tempo de vida	alzheimer's life span
alzheimer precoce	early alzheimer's
**Meditação**	**Meditation**
meditação ansiedade	anxiety meditation
meditação budista	buddhist meditation
meditação beneficios	meditation benefits
meditação youtube	youtube meditation
meditação musica	music meditation
meditação mindfulness	mindfulness meditation
meditação guiada	guided meditation
meditação app	meditation app
**Hipnose**	**Hypnosis**
hipnose como funciona	hypnosis how it works
para que serve a hipnose	what is hypnosis for
hipnose como fazer	hypnosis how to do
hipnose tecnicas	technical hypnosis
hipnose curso	hypnosis course
hipnose livro	hypnosis book
hipnose parapsicologia	parapsychology hypnosis
hipnose memória	memory hypnosis
**inteligencia artificial**	**artificial intelligence**
como funciona a inteligencia artificial	how artificial intelligence works
como surgiu a inteligencia artificial	how artificial intelligence came about
inteligencia artificial historia	artificial intelligence history
tipos de inteligencia artificial	types of artificial intelligence
inteligencia artificial google	google artificial intelligence
inteligencia artificial pdf	artificial intelligence pdf
inteligencia artificial filme	artificial intelligence movie
inteligencia artificial curso	artificial intelligence course
**Parkinson**	**Parkinson**
parkinson tratamento	parkinson treatment
parkinson fisiopatologia	parkinson pathophysiology
parkinson causas	parkinson causes
parkinson precoce	early parkinson
mal de parkinson sintomas finais	parkinson's disease final symptoms
parkinson prevenção	parkinson prevention

parkinson em jovens	parkinson in young
parkinson pdf	parkinson pdf
**Neuropsicologia**	**Neuropsychology**
neuropsicologia curso	neuropsychology course
neuropsicologia infantil	childhood neuropsychology
neuropsicologia pdf	neuropsychology pdf
neuropsicologia neurociência	neuropsychology neuroscience
neuropsicologia artigos	neuropsychology articles
neuropsicologia goiania	neuropsychology goiania
neuropsicologia livro	neuropsychology book
pos graduação em neuropsicologia campinas	graduate in neuropsychology campinas
**transtorno bipolar**	**bipolar disorder**
transtorno bipolar afetivo	affective bipolar disorder
transtorno bipolar teste	bipolar disorder test
transtorno bipolar tipo 2	type 2 bipolar disorder
sintomas de transtorno bipolar	symptoms of bipolar disorder
tratamentos para transtorno bipolar	treatments for bipolar disorder
transtorno bipolar cid	bipolar disorder cid
transtorno bipolar dsm v	bipolar disorder dsm v
transtorno bipolar é degenerativo	bipolar disorder is degenerative
**AVC**	**Stroke**
avc pdf	avc pdf
avc hemorragico	hemorrhagic stroke
avc sequelas	stroke sequels
avc isquemico	ischemic stroke
avc cuidados de enfermagem	stroke nursing care
como evitar um avc	how to avoid a stroke
epidemiologia avc brasil 2018	epidemiology avc brazil 2018
avc tem cura	stroke has cure
**Esquizofrenia**	**Schizophrenia**
esquizofrenia tipos	schizophrenia types
esquizofrenia simples	simple schizophrenia
esquizofrenia causas	schizophrenia causes
esquizofrenia catatonica	catatonic schizophrenia
esquizofrenia hebefrênica	hebephrenic schizophrenia
esquizofrenia indiferenciada	undifferentiated schizophrenia
esquizofrenia paranoide	paranoid schizophrenia
esquizofrenia tratamento	schizophrenia treatment

Sonhos	Dreams
baixar livro dos sonhos	download dream book
significado dos sonhos morte	meaning of death dreams
significado dos sonhos gravidez	meaning of pregnancy dreams
significado dos sonhos cobra	meaning of snake dreams
livro dos sonhos da vovó	grandma's dream book
significado dos sonhos joao bidu	meaning of dreams joao bidu
significado dos sonhos traição	meaning of dreams betrayal
interpretação de sonhos evangelicos	interpretation of evangelical dreams

Células-tronco	Stem cells
celulas tronco wikipedia	stem cells wikipedia
celulas tronco embrionarias	embryonic stem cells
importancia das celulas tronco	importance of stem cells
onde são encontradas as celulas tronco	where stem cells are found
celulas tronco pdf	stem cells pdf
celulas tronco polemica	controversial stem cells
celulas tronco filosofia	stem cells philosophy
embriologia celulas tronco	stem cell embryology

Eletroencefalograma	Electroencephalogram
eletroencefalograma para que serve	electroencephalogram
eletroencefalograma resultados	electroencephalogram results
eletroencefalograma preço	electroencephalogram Price
eletroencefalograma onde fazer	electroencephalogram where to do
laudo de eletroencefalograma normal	normal electroencephalogram report
eletroencefalograma sigla	electroencephalogram acronym
eletroencefalograma com mapeamento cerebral	electroencephalogram with brain mapping
resultado eletroencefalograma epilepsia	electroencephalogram result epilepsy

bulimia	bulimia
bulimía resumo	bulimia summary
anorexía resumo	anorexia summary
vigorexia	vigorexia
o que leva uma pessoa a tornar-se bulímica ou anoréxica	what causes a person to become bulimic or anorexic
transtornos alimentares	eating disorders
transtorno de compulsão alimentar	binge eating disorder
bulimía nervosa	nervous bulimia
disturbios alimentares	eating disorders

**Aneurisma**	**Aneurysm**
aneurisma abdominal	abdominal aneurysm
aneurisma cerebral causas	cerebral aneurysm causes
aneurisma cerebral sequelas	cerebral aneurysm sequelae
aneurisma cerebral fotos	cerebral aneurysm photos
aneurisma cerebral sintomas fotos	cerebral aneurysm symptoms pictures
aneurisma sintomas	aneurysm symptoms
cirurgia aneurisma cerebral recuperação	cerebral aneurysm recovery surgery
aneurisma significado	aneurysm meaning

**Epilepsia**	**Epilepsy**
epilepsia é contagiosa	epilepsy is contagious
epilepsia e convulsão	epilepsy and seizure
epilepsia tipos	epilepsy types
sintomas de epilepsia	epilepsy symptoms
epilepsia pega	epilepsy catches
epilepsia infantil	childhood epilepsy
epilepsia é hereditário	epilepsy is hereditary
epilepsia o que fazer	epilepsy what to do

**GABA**	**GABA**
gaba dosagem	gaba dosage
gaba farmacologia	gaba pharmacology
gaba bula	boast bull
gaba ansiedade	boasts anxiety
gaba receptor	gaba receiver
gaba e melatonina	gaba and melatonin
gaba pdf	gaba pdf
neurotransmissor gaba pdf	neurotransmitter gaba pdf

**Esclerose Múltipla**	**Multiple sclerosis**
esclerose multipla sintomas	multiple sclerosis symptoms
esclerose multipla diagnostico	multiple sclerosis diagnosis
esclerose multipla tempo de vida	lifetime multiple sclerosis
esclerose multipla pode matar	multiple sclerosis can kill
esclerose multipla fisiopatologia	multiple sclerosis pathophysiology
esclerose multipla expectativa de vida	multiple sclerosis life expectancy
esclerose multipla é hereditaria	multiple sclerosis is hereditary
esclerose multipla prevenção	multiple sclerosis prevention

**Serotonina**	**Serotonin**

serotonina como aumentar	serotonin how to increase
serotonina baixa	low serotonin
serotonina alta	high serotonin
serotonina exame	serotonin exam
serotonina comprar	serotonin buy
serotonina neurotransmissor	neurotransmitter serotonin
serotonina e dopamina	serotonin and dopamine
serotonina função	serotonin function

**Doppler**	**Doppler**
exame doppler gravidez	pregnancy doppler exam
doppler venoso	venous doppler
doppler colorido	color doppler
doppler obstetrico	obstetric doppler
ultrassom com doppler quando fazer	doppler ultrasound when doing
doppler de carotidas	carotid doppler
ultrassom com doppler tireoide	thyroid doppler ultrasound
ultrassom com doppler 32 semanas	doppler ultrasound 32 weeks

**TDAH**	**ADHD**
tdah tratamento	adhd treatment
tdah teste	adda test
tdah sintomas	adhd symptoms
tdah cid	adhd cid
tdah na escola	adda at school
tdah artigos	adhd articles
tdah pdf	adhd pdf
tdah caracteristicas	adhd features

**Hormônios**	**Hormones**
tipos de hormonios	types of hormones
para que serve os hormonios	what are hormones for
onde são produzidos os hormonios	where hormones are produced
tabela de hormonios e suas funções	hormone table and its functions
classificação dos hormonios	classification of hormones
hormonios femininos	female hormones
hormonios locais	local hormones
trabalho sobre hormonios	hormone work

**Síndrome do Pânico**	**Panic Syndrome**
sindrome do panico teste	panic test syndrome

sindrome do panico tratamento	panic syndrome treatment
sindrome do panico sintomas diarios	panic syndrome daily symptoms
síndrome do pânico tem cura	panic syndrome has a cure
sindrome do panico pode matar	panic syndrome can kill
sindrome do panico sintomas mentais	panico syndrome mental symptoms
sindrome do panico cura sozinha	panic syndrome cures alone
nomes de remedios para sindrome do panico	names of remedies for panic syndrome

**Síndrome do Pânico**	**Panic Syndrome**
sindrome do panico teste	panic test syndrome
sindrome do panico tratamento	panic syndrome treatment
sindrome do panico sintomas diarios	panic syndrome daily symptoms
síndrome do pânico tem cura	panic syndrome has a cure
sindrome do panico pode matar	panic syndrome can kill
sindrome do panico sintomas mentais	panico syndrome mental symptoms
sindrome do panico cura sozinha	panic syndrome cures alone
nomes de remedios para sindrome do panico	names of remedies for panic syndrome

**cafeína**	**caffeine**
cafeína suplemento	caffeine supplement
cafeína 420mg	420mg caffeine
cafeína benefícios	caffeine benefits
cafeína cápsula	caffeine capsule
cafeína molécula	caffeine molecule
cafeína café	caffeine coffee
cafeína efeitos	caffeine effects
cafeína fórmula	caffeine formula

S3 Table. Principal components analysis showing the independent (categorical variables) are unrelated. All *ps* > 0.05.

	Age	Region	Profession
Age	1.0000	-0.1292	0.1074
Region	-0.1292	1.0000	0.1283
Profession	0.1074	0.1283	1.000

S4 Table. Age x Score. Age groups: 10-19 years old, and so on; N: number of participants in each group; Mean (SEM): mean scores for each group (standard error of the mean).

Age	N	Mean (SEM)
10-19	41	0.71 (0.0182)
20-29	266	0.72 (0.0055)
30-39	380	0.71 (0.0048)
40-49	236	0.70 (0.0059)
50-59	137	0.69 (0.0076)
60+	68	0.66 (0.0117)

S5 Table. Age x Score. Significant post-hoc Tukey HSD tests for age; 1 = 10-19 year-olds; 2 = 20-29 year-old group, 3 = 30-39 group, 4 = 40-49 group, 5 = 50-59 group, 6 = 60 and older group.

Tukey HSD	p-value
6 x 4	0.0283
6 x 3	0.0001
6 x 2	0.0001
5 x 2	0.0249

S6 Table. Region x Score. Region groups: South, Southeast, Midwest, North, Northeast and Foreign. N: number of participants in each group; Mean (SEM): mean scores for each group (standard error of the mean).

Region	N	Mean (SEM)
South	109	0.70 (0.0091)
Southeast	756	0.71 (0.0034)
Midwest	41	0.71 (0.0131)
North	44	0.68 (0.0160)
Northeast	154	0.69 (0.0072)

S7 Table. Profession x Score. Profession groups: Bio (biological sciences), Exa (exact sciences), Hum (humanities), Hea (health sciences), and Other ('other', retired, or not working); N: number of participants in each group; Mean (SEM): mean scores for each group (standard error of the mean).

Prof	N	Mean (SEM)
Bio	92	0.76 (0.0096)
Exa	104	0.71 (0.0096)
Hum	104	0.71 (0.0044)
Hea	307	0.72 (0.0050)

**Other**	**220**	**0.66 (0.0062)**

S8 Table. Profession x Score. Significant post-hoc Tukey HSD tests for profession. The Bio group performed significantly better than all other groups, while the other three groups with a defined profession (Exa, Hum and Hea) only performed significantly better than the 'Other' group but did not differ from each other.

**Tukey HSD**	**p-value**
**Bio x Exa**	**0.0001**
**Bio x Hum**	**0.0001**
**Bio x Hea**	**0.0001**
**Bio x Other**	**0.0001**
**Exa x Other**	**0.0003**
**Hum x Other**	**0.0001**
**Hea x Other**	**0.0001**

S9 Table. Southeast, Age. ANOVA

**Age**	**N**	**Mean (SEM)**
**10-19**	**21**	**0.73 (0.0239)**
**20-29**	**150**	**0.73 (0.0077)**
**30-39**	**261**	**0.72 (0.006)**
**40-49**	**166**	**0.71 (0.007)**
**50-59**	**110**	**0.70 (0.0081)**
**60+**	**48**	**0.68 (0.0123)**

S10 Table. Southeast, Age. Post-hoc Tukey HSD tests.

**Tukey HSD**	**p-value**
**6 x 3**	**0.0357**
**6 x 2**	**0.0114**

S11 Table. Midwest, Age. ANOVA

**Age**	**N**	**Mean (SEM)**
**20-29**	**7**	**0.75 (0.0176)**
**30-39**	**15**	**0.74 (0.0179)**
**40-49**	**8**	**0.70 (0.0357)**
**50-59**	**6**	**0.70 (0.0358)**
**60+**	**5**	**0.60 (0.0194)**

S12 Table. Midwest, Age. Post-hoc Tukey HSD tests.

**Tukey HSD**	**p-value**
**6 x 3**	**0.0124**
**6 x 2**	**0.0125**

S13 Table. Northeast, Age. ANOVA

Age	N	Mean (SEM)
10-19	15	0.66 (0.0275)
20-29	60	0.71 (0.0094)
30-39	40	0.70 (0.0134)
40-49	28	0.70 (0.0162)
50-59	6	0.61 (0.0355)
60+	5	0.57 (0.0614)

S14 Table. Northeast, Age. Post-hoc Tukey HSD tests.

Tukey HSD	p-value
6 x 4	0.0229
6 x 3	0.0252
6 x 2	0.0139

S15 Table. Southeast, Profession. ANOVA

Prof	N	Mean (SEM)
Bio	68	0.77 (0.0104)
Exa	80	0.70 (0.0114)
Hum	302	0.71 (0.0049)
Hea	175	0.72 (0.0071)
Other	131	0.68 (0.0080)

S16 Table. Southeast, Profession. Post-hoc Tukey HSD tests.

Tukey HSD	p-value
Other x Bio	0.0001
Other x Hum	0.0034
Other x Hea	0.0001
Bio x Exa	0.0001
Bio x Hum	0.0001
Bio x Hea	0.0010

S17 Table. Northeast, Profession. ANOVA

Prof	N	Mean (SEM)
Bio	7	0.75 (0.0439)
Exa	10	0.70 (0.0308)
Hum	25	0.68 (0.0192)
Hea	81	0.71 (0.0085)
Other	31	0.64 (0.0155)

S18 Table. Northeast, Profession. Post-hoc Tukey HSD tests.

Tukey HSD	p-value
Other x Bio	0.0251
Other x Hea	0.0021

S19 Table. South, Profession. ANOVA

Prof	N	Mean (SEM)
Bio	5	0.69 (0.0378)
Exa	9	0.75 (0.0253)
Hum	44	0.71 (0.0150)
Hea	26	0.71 (0.0154)
Other	25	0.65 (0.0191)

S20 Media vs. Neuromyths: Age, means and SEMs

Age	N	Media – mean (SEM)	Neuromyth – mean (SEM)
10-19	41	0.81 (0.0150)	0.57 (0.0240)
20-29	266	0.83 (0.0059)	0.58 (0.0086)
30-39	380	0.83 (0.0050)	0.58 (0.0071)
40-49	236	0.83 (0.0065)	0.54 (0.0093)
50-59	137	0.81 (0.0079)	0.54 (0.0113)
60+	68	0.80 (0.0155)	0.51 (0.0144)

S21 Media vs. Neuromyths: Region, means and SEMs

Region	N	Media – mean (SEM)	Neuromyth – mean (SEM)
South	109	0.80 (0.0096)	0.58 (0.0136)
Southeast	756	0.83 (0.0036)	0.57 (0.0051)
Midwest	41	0.80 (0.0161)	0.58 (0.0188)
North	44	0.80 (0.0204)	0.53 (0.0220)
Northeast	154	0.82 (0.0080)	0.54 (0.0108)

S22 Media vs. Neuromyths: Profession, means and SEMs

Prof	N	Media – mean (SEM)	Neuromyth – mean (SEM)
Bio	92	0.85 (0.0093)	0.64 (0.0151)
Exa	104	0.82 (0.0090)	0.57 (0.0144)
Hum	104	0.83 (0.0048)	0.57 (0.0066)
Hea	307	0.83 (0.0055)	0.57 (0.0078)
Other	220	0.81 (0.0076)	0.50 (0.0088)
