# Supplementary material for: What Does the General Public Know (or Not) About Neuroscience? Effects of Age, Region and Profession in Brazil
Source: Front Hum Neurosci. 2022 Mar 4;16:798967. doi: 10.3389/fnhum.2022.798967 (PMC8930840; doi:10.3389/fnhum.2022.798967)
Supplement: Supplementary file 2 [file Image_1.pdf]

Respostas da pesquisa realizada em Setembro/Outubro de 2019

DESVENDANDO MITOS DAS NEUROCIÊNCIAS

Quer saber as respostas corretas sobre os maiores mitos ligados ao funcionamento do cérebro e do sistema nervoso? Então você está no lugar certo.

Desvendando mitos das Neurociências

1. Apesar de pesar em média 1,2kg e ter entre 80 e 100 bilhões de neurônios, só utilizamos 10% da capacidade do nosso cérebro.

RESPOSTA CORRETA

	Verdadeiro	Falso
Respostas ao questionário	56%	44%

FALSO - Apesar de ser um mito bastante difundido, aparecendo inclusive em séries e filmes, utilizamos 100% da capacidade do nosso cérebro. O cérebro humano adulto pesa cerca de 1400g, aproximadamente 2% do peso corporal médio. Contém perto de 86 bilhões de neurônios, componentes básicos que criam uma intricada arquitetura de conexões sinápticas (sinapses) para o processamento de informação e geração de respostas. Em média, um único neurônio forma e recebe de 1000 a 10000 contatos. Desse modo, no cérebro chegam a se formar uma totalidade de 10ˆ14 a 10ˆ15 conexões sinápticas. Portanto, existem 1000 vezes mais sinapses no cérebro humano do que as 100 bilhões de estrelas da galáxia.

Quando deixamos de usar alguma célula nervosa, ela morre. Em um exame de ressonância magnética funcional, podemos ver que qualquer comando, por mais simples que seja, ativa muito mais áreas do que cabem nesses 10%. Além disso, existem redes amplas de ativação mesmo enquanto achamos que “não estamos pensando em nada” ou não focando em nada em particular, tais como o Default Mode Network e o Resting State Network.

Fonte: von Bartheld CS, Bahney J, Herculano-Houzel S. The search for true numbers of neurons and glial cells in the human brain: A review of 150 years of cell counting. J Comp Neurol . 2016;524:3865–95. Available from: http://www.ncbi.nlm.nih.gov/pubmed/27187682

Desvendando mitos das Neurociências

2. A doença de Alzheimer só pode ser diagnosticada após a morte. Em vida, podem ser identificados comportamentos através de testes neuropsicológicos que sugerem a presença da doença.

RESPOSTA CORRETA

	Verdadeiro	Falso
Respostas ao questionário	33%	67%

VERDADEIRO - A doença de Alzheimer se caracteriza pela degeneração e morte das células nervosas - os neurônios. Algumas das consequências são a perda progressiva da memória e da linguagem. Ainda não se conhece a causa, apesar da existência de uma série de hipóteses, desde fatores genéticos até o acúmulo de proteínas entre os tratos neurais. A única certeza: os exames disponíveis não possibilitam a confirmação do Alzheimer em vida. Isso porque essa degeneração e morte celular típicas do Alzheimer só podem ser vistas no cérebro dissecado. Em outras palavras, não há exame que comprove que um caso de demência é Alzheimer. Com isso, estima-se que mais de 10% dos casos diagnosticados são na verdade outros tipos de demência. Existem alguns avanços recentes oferecendo novas possibilidades de diagnóstico em vida, como usar marcadores radiativos em testes de neuroimagem (PET) e testes de conectividade (as comunicações entre redes neurais) para visualizar o acumulo de proteínas especificas (tau) no cérebro de pessoas com e sem a doença. Mas por enquanto, em vida, o melhor que se pode fazer é aplicar testes neuropsicológicos para se aproximar o máximo possível de um diagnóstico certeiro, ja que o mais importante é identificar e tratar o comportamento do paciente, independente do diagnostico.

Fonte: Maruyama M, Shimada H, Suhara T, Shinotoh H, Ji B, Maeda J, et al. Imaging of Tau Pathology in a Tauopathy Mouse Model and in Alzheimer Patients Compared to Normal Controls. Neuron . 2013 .79:1094–108. Available from: http://www.ncbi.nlm.nih.gov/pubmed/24050400

e - Franzmeier N, Rubinski A, Neitzel J, Kim Y, Damm A, Na DL, et al. Functional connectivity associated with tau levels in ageing, Alzheimer's, and small vessel disease. Brain . 2019 .142:1093–107. Available from: http://www.ncbi.nlm.nih.gov/pubmed/30770704

Desvendando mitos das Neurociências

3. As diferenças estruturais entre os cérebros masculinos e femininos são tão óbvias que qualquer profissional poderia identificar o sexo da pessoa simplesmente olhando uma imagem do cérebro dela.

RESPOSTA CORRETA

	Verdadeiro	Falso
Respostas ao questionário	17%	83%

FALSO - Muitos estudos tem identificado algumas diferenças estruturais e até funcionais entre os cérebros feminino e masculino. No entanto, em 2016, U. Dicke e G. Roth realizaram um estudo para entender os fatores envolvidos na inteligência, concluindo que as singularidades se encontram nas propriedades do cérebro e não nas proporções. As diferenças tendem a ser maiores entre membros do mesmo sexo que membros do sexo oposto (ou seja, as diferencas entre indivíduos, independente do sexo, são mais significativas).

Portanto é difícil identificar rasgos específicos de cada sexo. Ou seja, ninguém poderia pegar um exame de imagem de um cérebro e definir de qual sexo ou gênero uma pessoa é. Além disso, estudos que encontraram diferencas nas formas/estratégias de resolver problemas observam que as estratégias de um sexo podem ser facilmente treinadas e aprendidas por indivíduos do outro sexo, sugerindo que algumas tendencias cognitivas tem origens culturais/sociais e não biológicas.

Fonte: Dicke U, Roth G. Neuronal factors determining high intelligence . Philos. Trans. R. Soc. B Biol. Sci. 2016 [cited 2018 Aug 24]. Available from: http://dx.doi.org/10.1098/rstb.2015.0180

4. A meditação se dá no estado alfa - quando nossas ondas cerebrais nos possibilitam um relaxamento profundo

RESPOSTA CORRETA

	Verdadeiro	Falso
Respostas ao questionário	70%	30%

VERDADEIRO - O cérebro funciona através de estímulos elétricos e entra em vários estados - ou seja, as ondas eletromagnéticas utilizam diferentes frequências para realizar determinadas tarefas. No estado alfa, temos uma menor resposta aos estímulos externos, o que faz com que tenhamos maior concentração e relaxamento - excelentes para a meditação!

Fonte: Lagopoulos J, Xu J, Rasmussen I, Vik A, Malhi GS, Eliassen CF, et al. Increased Theta and Alpha EEG Activity During Nondirective Meditation. J Altern Complement Med . 2009 .15:1187–92. Available from: http://www.ncbi.nlm.nih.gov/pubmed/19922249

RESPOSTA CORRETA

5. A serotonina é um medicamento para depressão produzido somente no laboratório.

	Verdadeiro	Falso
Respostas ao questionário	5%	95%

FALSO - A serotonina é um neurotransmissor produzido pelo próprio corpo. Um neurotransmissor "leva informações" de um neurônio a outro. A serotonina responde por uma série de processos no cérebro, como o humor, a fome, a agressividade, sono e temperatura corporal. Os medicamentos existentes (como antidepressivos) não possuem serotonina, mas sim ajudam a regular os níveis da serotonina já existente no organismo do indivíduo.

6. O número absoluto de neurônios determina o poder da nossa memória e cognição em geral.

RESPOSTA CORRETA

	Verdadeiro	Falso
Respostas ao questionário	25%	75%

FALSO - Como abordado na pregunta 1, o cérebro contém uma média de 86 bilhões de neurônios. O poder de nossa memória e cognição estão ligados ao uso, que cria e fortalece as conexões necessárias para a realização dessas tarefas. Por tanto, a memória e a cognição não se determinam pelo número de neurônios, mas sim pelas conexões adequadas e aperfeiçoadas para uma função determinada (tendo também em vista que maior número de conexões não é o que precisamos, se não conexões funcionais e específicas).

7. A ansiedade é causada por distúrbios químicos no cérebro

RESPOSTA CORRETA

	Verdadeiro	Falso
Respostas ao questionário	70%	30%

VERDADEIRO - Pelo ponto de vista das Neurociências, a ansiedade é causada por um distúrbio químico no cérebro. Áreas ligadas à emoção e tomada de ação/decisão se colocam em alerta e trocam informações para preparar o corpo para preparar o corpo para lidar com situações de emergência ou perigo. Quando isso acontece sem a existência de um perigo real, esse desequilíbrio químico perene se torna debilitante para a vida regular do indivíduo.

Em outras matérias correlatas, os cientistas já são capazes de trilhar algumas razões para esse desequilíbrio, podendo ser de traumas psicológicos ou físicos, por exemplo. Por isso, o tratamento é multidisciplinar, exigindo não apenas a correção desse desbalanço químico quanto a observação das matrizes causais.

8. Cada neurônio armazena uma informação diferente.

RESPOSTA CORRETA

Respostas ao questionário	Verdadeiro	Falso
	43%	57%

FALSO - As informações em nosso cérebro são resultado de um conjunto de comandos armazenados em diversos neurônios, que se combinam para criar novos direcionamentos. Um mesmo neurônio pode estar envolvido em diversas tarefas.

9. Usamos o cérebro as 24h do dia.

RESPOSTA CORRETA

	Verdadeiro	Falso
Respostas ao questionário	93%	7%

VERDADEIRO - Mesmo dormindo, nosso cérebro continua em funcionamento. É como o coração ou qualquer outro órgão do corpo: está o tempo todo fazendo nosso organismo funcionar corretamente. Como ele é responsável por todas as informações que correm no corpo, até mesmo em estado de inconsciência, há uma regulação ainda que o cérebro deve fazer. Se o cérebro parasse de funcionar esporadicamente, não seria possível a manutenção da vida.

10. O exame de Ressonância Magnética pode ser utilizado para saber o que as pessoas pensam

RESPOSTA CORRETA

	Verdadeiro	Falso
Respostas ao questionário	6%	94%

FALSO - O exame de Ressonância Magnética, em geral, serve para fazer uma "imagem" de como a estrutura e a fisiologia do corpo estão dispostas. Mesmo numa análise de ressonância magnética funcional, só' se pode inferir se o sujeito estava fazendo a tarefa solicitada ou não (o que nunca é uma garantia, já que poderia estar distraído). Interpretar os pensamentos de alguém 'do nada' é, até agora, algo que se vê somente em filmes de ciência ficção.

11. Há períodos críticos ou sensíveis na infância após os quais certas coisas ficam mais difíceis de aprender

RESPOSTA CORRETA

Respostas ao questionário	Verdadeiro	Falso
	64%	37%

VERDADEIRO - Durante o começo da vida, a plasticidade do nosso cérebro está no auge. Isto significa que ele é muito sensível aos eventos internos e externos que irão modificar a sua estrutura e funcionalidade. O nosso cérebro possui plasticidade ao longo da vida, mas geralmente a sua intensidade diminui progressivamente. Portanto, algumas coisas ficam mais difíceis de aprender depois dos períodos de maior plasticidade que ocorrem bem cedo. Além disso, diferentes sistemas (visão, linguagem, audição) possuem períodos 'críticos' ou 'sensíveis' diferentes.

12. O ser humano pode sofrer Esclerose Múltipla em qualquer momento da vida

RESPOSTA CORRETA

Respostas ao questionário	Verdadeiro	Falso
	82%	18%

VERDADEIRO - A Esclerose Múltipla é uma doença autoimune (quando o corpo ataca a uma parte de si mesmo como uma ameaça). Nela, há uma desmielinização dos tratos neurais.

A mielina é uma gordura que reveste uma parte do neurônio - o axônio - responsável por conduzir os impulsos elétricos. Essa gordura é altamente condutora. Sem ela, as mensagens entre os neurônios ficam comprometidas, levando a diversos estados de dificuldades sensitivas e motoras. Isso pode acontecer em qualquer momento da vida, sendo inclusive muito comum em pessoas entre 20 e 30 anos.

13. Todos os pacientes que sofrem AVC perdem a fala

RESPOSTA CORRETA

Respostas ao questionário	Verdadeiro	Falso
	1%	99%

FALSO - Nem todos. O Acidente Vascular Cerebral (AVC) pode acontecer em qualquer área do cérebro, prejudicando assim as funções relativas às estruturas acometidas. Por isso, a fala pode ser perdida, mas não é uma regra. A maior importância dessa informação reside em oferecer mais e melhores ferramentas para que as pessoas identifiquem corretamente um AVC, de forma a não ignorar sintomas importantes só porque a fala permanece intacta.

14. As drogas não alteram a composição bioquímica do cérebro, mas alteram o comportamento

RESPOSTA CORRETA

Respostas ao questionário	Verdadeiro	Falso
	26%	74%

FALSO - Além de alterar o comportamento durante seu uso, as drogas também alteram a fisiologia do cérebro. Um exemplo é o do sistema dopaminérgico: as drogas estimulam o sistema de recompensa do indivíduo, e o cérebro passa a entendê-las como mais benéficas do que prejudiciais. Isso depende de uma alteração bioquímica.

Ainda assim, muitas alterações podem ser revertidas - dentro dos parâmetros corretos e com o acompanhamento de profissionais preparados para lidar com esse tipo de caso.

15. A morte celular na doença de Parkinson causa sintomas motores como congelamento ou tremores.

RESPOSTA CORRETA

Respostas ao questionário	Verdadeiro	Falso
	90%	10%

VERDADEIRO - A doença de Parkinson se caracteriza pela morte celular dos neurônios que produzem a Dopamina. Este, por sua vez, é um neurotransmissor responsável tanto pelo sistema de recompensa do cérebro quanto pelos movimentos motores do corpo. Com a ausência de sua produção, é comum que os portadores da doença de Parkinson apresentem quadros de congelamentos e tremores.

16. As vacinas causam autismo em crianças em desenvolvimento

RESPOSTA CORRETA

	Verdadeiro	Falso
Respostas ao questionário	2%	98%

FALSO - O autismo é um distúrbio de desenvolvimento. Seus portadores são categorizados dentro de um espectro, que pode ir do mais leve ao mais grave. Esse distúrbio não está ligado a vacinas. Esse mito começou em 1998, quando Andrew Wakefield, um gastroenterologista (agora ex e desacreditado) publicou um estudo alegando uma conexão entre a vacina tríplice (sarampo, rubéola e caxumba) e o autismo. Pouco tempo depois, o estudo foi rejeitado pela revista por ter apresentado dados falsos, e em 2004, descobriu-se que o Wakefield tinha conflitos de interesse financeiros na hora de publicar o estudo. Ao mesmo tempo, uma atriz e outras pessoas da mídia divulgaram histórias pessoais de filhos com autismo desenvolvido logo após ter tomado a vacina. Uma dessas atrizes americanas apareceu pouco tempo depois afirmando que o filho de fato não tinha autismo.

Não existe nenhum link cientificamente comprovado entre vacinas e autismo, e por mais que os cientistas tenham tentado ‘consertar’ esses erros divulgados tão amplamente, o dano já havia sido feito. Uma pessoa não vacinada pode conduzir doenças que já haviam sido erradicadas, o que tem acontecido em várias partes do mundo nos últimos anos, por motivos de religião e também a falsa ideia da conexão entre as vacinas e o autismo. Então a vacina não deve ser apenas uma decisão pessoal, mas coletiva.

17. Apesar de só lembrarmos pequenas partes ou recortes dos sonhos, eles são longos e acontecem em tempo real em relação aos seus eventos.

RESPOSTA CORRETA

	Verdadeiro	Falso
Respostas ao questionário	42%	58%

VERDADEIRO - É comum lembrarmos de trechos desconexos dos sonhos, mas eles acontecem "em tempo real" em relação a seus acontecimentos.

Se você sonha que foi até a padaria, por exemplo, em sua mente a experiência inteira foi vivida, mesmo que de manhã você só se lembre do trecho de "saindo de casa" e depois "um diálogo estranho na padaria".

Fonte: Erlacher D, Schädlich M, Stumbrys T, Schredl M. Time for actions in lucid dreams: effects of task modality, length, and complexity. Front Psychol . Frontiers; 2014 .4:1013. Available from: http://journal.frontiersin.org/article/10.3389/fpsyg.2013.01013/abstract

18. Cada região do cérebro tem uma função única.

RESPOSTA CORRETA

Respostas ao questionário	Verdadeiro	Falso
	53%	47%

FALSO - Assim como respondemos na questão 8, nem os neurônios têm função única, nem as regiões do cérebro. Todas trabalham em diversas frentes. Podemos aferir que algumas regiões são "mais responsáveis por" ou “o centro de” determinadas funções, mas geralmente é uma série de estruturas envolvidas em uma única função.

19. A Neuroplasticidade, capacidade do sistema nervoso de mudar e adaptar-se, termina após a adolescência

RESPOSTA CORRETA

Respostas ao questionário	Verdadeiro	Falso
	14%	86%

FALSO - Qualquer novo aprendizado requer uma nova conexão cerebral. Isso significa que todos os dias, o tempo todo, o cérebro está se ajustando e se adaptando para corresponder aos estímulos que nos são apresentados e para poder responder a eles. Como explicado na questão 11, a plasticidade pode diminuir ao longo do tempo, mas com certeza continua ao longo da vida.

20. Os humanos são os únicos seres vivos que possuem consciência

RESPOSTA CORRETA

	Verdadeiro	Falso
Respostas ao questionário	45%	55%

FALSO - Estudos mostram que os animais não-humanos - tais como cães, gatos, polvos, macacos ou elefantes - produzem sinais cerebrais de alta semelhança com os humanos, inclusive no que tange à existência de uma consciência. Portanto, não é mais correto dizer que só os humanos possuem essa instância mental, mesmo que esta difira do que conhecemos como "consciência humana".

21. A imaginação pode criar memórias falsas, acontecimentos que acreditamos ter vivenciado mas nunca aconteceram

RESPOSTA CORRETA

Respostas ao questionário	Verdadeiro	Falso
	94%	6%

VERDADEIRO - A memória também passa por processos de adaptação. Ela vai se moldando de acordo com as experiências às quais o indivíduo é submetido. Elas servem para nos dar subsídio para a tomada de decisão (consciente ou inconsciente). Como elas são gravadas a partir de comandos emocionais (as áreas da emoção no cérebro entendem que aquela é uma memória importante e a consolida), novos processos emocionais - desde um desejo até uma história contada por um parente de confiança - podem alterar a memória que já estava ali. Uma das preocupações em relação a esse fato está ligada ao campo jurídico, já que em uma prestação de depoimento, por exemplo, uma pessoa pode ser conduzida a lembrar de fatos que não aconteceram.

22. Cérebros de maior tamanho são mais inteligentes

RESPOSTA CORRETA

Verdadeiro | Falso

Respostas ao questionário: 7% | 93%

FALSO - Assim como falamos na resposta à pergunta 6, não são os fatores estruturais que determinam inteligência ou cognição, mas a forma como as conexões estão estruturadas e são estimuladas. Assim, um cérebro maior não faz da pessoa mais inteligente.

23. A melhor prevenção da doença de Alzheimer é fazer exercício físico.

RESPOSTA CORRETA

Verdadeiro | Falso

Respostas ao questionário: 51% | 49%

VERDADEIRO - A leitura e o exercício da cognição são de suma importância para a manutenção do cérebro, assim como dormir bem. Ainda assim, as substâncias produzidas pelo corpo através do exercício físico também têm efeitos sobre o cérebro. Um exemplo é o da Irisina, um hormônio descoberto recentemente e que age como um “protetor neural” - sendo liberada em maior quantidade naqueles que praticam exercícios físicos regularmente. O exercício físico também atua nos vasos sanguíneos, na pressão sanguínea e no fortalecimento de estruturas cerebrais irrigadas por esses vasos. Portanto, ler é bom, mas ate agora o link mais forte é entre saúde cognitiva e exercício físico.

Fonte: Erlacher D, Schädlich M, Stumbrys T, Schredl M. Time for actions in lucid dreams: effects of task modality, length, and complexity. Front Psychol . Frontiers; 2014 .4:1013. Available from: http://journal.frontiersin.org/article/10.3389/fpsyg.2013.01013/abstract

24. Durante o sono, nossa atividade cerebral diminui

RESPOSTA CORRETA

Respostas ao questionário	Verdadeiro	Falso
	63%	37%

FALSO - Na realidade, o que acontece durante o sono é uma mudança das ondas cerebrais, que vão desde o relaxamento inicial até um estágio de sono mais profundo. Mas o cérebro continua em atividade normalmente e a todo vapor.

25. As pontuações de QI podem mudar com o tempo.

RESPOSTA CORRETA

Respostas ao questionário	Verdadeiro	Falso
	83%	17%

VERDADEIRO - Os testes de QI se prestam a avaliar a capacidade cognitiva de um indivíduo. Mas a capacidade cognitiva é flutuante: como o cérebro tem a habilidade de adaptação de acordo com novas experiências e aprendizados, o resultado do teste de QI provavelmente apresentará divergências se realizados pela mesma pessoa em momentos cronológicos diferentes.

26. Quando vemos cores diferentes em um vestido ou tênis, é por que estamos usando o lado dominante de nosso cérebro (direito ou esquerdo)

RESPOSTA CORRETA

	Verdadeiro	Falso
Respostas ao questionário	61%	39%

FALSO - Os lados do cérebro não interferem nas cores que vemos - ou pelo menos, não dessa forma pragmática. Quando vemos cores diferentes em um objeto (como foi a moda do tênis e do vestido com cores diferentes), o que temos é uma mistura de fatores que vai desde a iluminação do ambiente e da tela até a forma como o nervo óptico entende a informação apresentada. Mas novamente: nenhuma conexão com o lado do cérebro (inclusive porque usamos os dois ao mesmo tempo, o tempo todo!).

Rabin J, Houser B, Talbert C, Patel R. Blue-Black or White-Gold? Early Stage Processing and the Color of 'The Dress' 2016 Available from: https://journals.plos.org/plosone/article?id=10.1371/journal.pone.0161090

Desvendando mitos das Neurociências

27. O período entre 0 e 3 anos de idade é um período de crescimento e proliferação neuronal muito importante. Para um melhor desempenho na vida, a criança deve ser exposta a todos os estímulos possíveis durante este período, tais como matemática, linguagem e música.

RESPOSTA CORRETA

	Verdadeiro	Falso
Respostas ao questionário	75%	25%

FALSO - Nesse período (dos 0 aos 3 anos), o cérebro está muito propenso a aprender novas coisas. Mas nem todos os substratos neurais envolvidos em determinadas tarefas - como linguagem - se encontram completamente desenvolvidos. Desta forma, não adianta expor sistematicamente uma criança a esses estímulos esperando que assim ela se torne um futuro "Mozart" ou "Einstein". A criança deve ser estimulada sempre, mas cada tarefa dentro de seu tempo.

28. A epilepsia não é contagiosa, apesar de poder ser hereditária

RESPOSTA CORRETA

	Verdadeiro	Falso
Respostas ao questionário	92%	8%

VERDADEIRO - Não é possível transmitir a epilepsia, que se caracteriza pela perda de consciência e convulsões em intervalos regulares de tempo. Mas estudos já mostram que em muitos casos, o fator genético possui uma preponderância. Assim, o filho de um epiléptico terá maior probabilidade de apresentar a doença também.

29. A criança em desenvolvimento precisa de muitos estímulos constantes para aprender melhor. Por isso, é positivo o uso de tablet ou celular nos primeiros anos de vida.

RESPOSTA CORRETA

Respostas ao questionário	Verdadeiro	Falso
	23%	77%

FALSO - De fato, estimular a criança é uma excelente forma de promover seu desenvolvimento. Porém, telas em geral - TV, tablet ou celular - pode desencadear um atraso no desenvolvimento se consumidas em excesso. Alguns cientistas apontam que a criança, quando focada no aparelho, perde os estímulos ambientais que estão ocorrendo ao redor, além de prejudicar sua interação social, a qual deve ser desenvolvida na infância. Por isso: sim, a criança pode usar a tela como entretenimento, mas é altamente recomendável evitar o uso nos primeiros anos de vida ou excessos ao longo do crescimento.

30. Em estado de hipnose, perdemos completamente a consciência

RESPOSTA CORRETA

	Verdadeiro	Falso
Respostas ao questionário	27%	73%

FALSO - Durante a hipnose, o que ocorre é uma "quebra" de diálogo entre o sistema emocional (límbico) e o consciente (cortical). Desta forma, a pessoa hipnotizada vê tudo, compreende tudo e fica o tempo todo consciente - mas muito mais vulnerável às instruções passadas, já que não conta com seu sistema límbico respondendo aos estímulos. Com a inibição de sentimentos como dor, vergonha, medo, iniciativa ou prazer (por exemplo), o hipnotizado fica sem reservas ou referências, se colocando assim num papel de alta suscetibilidade.
